# Substituted 1,3,5-Triazine Hexacarboxylates as Potential Linkers for MOFs

**DOI:** 10.3390/molecules24193480

**Published:** 2019-09-25

**Authors:** Arne Klinkebiel, Ole Beyer, Ulrich Lüning

**Affiliations:** Otto-Diels-Institut für Organische Chemie der Christian-Albrechts-Universität zu Kiel, D-24098 Kiel, Germany; aklinkebiel@oc.uni-kiel.de (A.K.); obeyer@oc.uni-kiel.de (O.B.)

**Keywords:** ligands, metal organic framework, Sonogashira reaction, Suzuki reaction, triazine

## Abstract

Hexacarboxylates are promising linkers for MOFs such as NU-109 or NU-110, which possess large values for surfaces and pore volumina. Starting from 2,4,6-tris(bromoaryl)-1,3,5-triazines, palladium-catalyzed cross coupling reactions (Suzuki-Miyaura, Sonogashira-Hagihara) form elongated hexacarboxylate linkers. Eight new 2,4,6-tris(biphenyl) and 2,4,6-tris(phenylethynylphenyl) 1,3,5-triazines have been prepared in quantities ranging from 40 mg to 1.1 g. In five cases, one of the arms of the linker carries an additional functionality (NO_2_ or OMe).

## 1. Introduction

Porous metal-organic frameworks (MOFs) [1,2,3,4] are interesting materials for many applications, for instance gas absorption and storage. Large surfaces and pore volumes have been sought after, and excellent results were obtained when hexadendate linkers were employed in the syntheses of MOFs [5,6,7,8,9,10,11]. Remarkable values for surfaces and pore volumina have been measured for NU-109 and NU-110 (>7000 m^2^/g, 4 cm^3^/g) [10]. In these MOFs, hexadentate linkers based on 1,3,5-trisubstituted benzenes have been used, which carry isophthalic acids at the end of each substituent.

The properties of MOFs can be altered by the introduction of additional substituents. There are two general approaches: substituted linkers can be used, or the additional functionality is introduced post-synthetically. However, post-synthetic modifications are rarely quantitative and are frequently accompanied by decomposition especially when the material possesses a high porosity. Therefore, the use of already functionalized linkers will lead to more homogeneous MOFs. 

**Figure 1:**
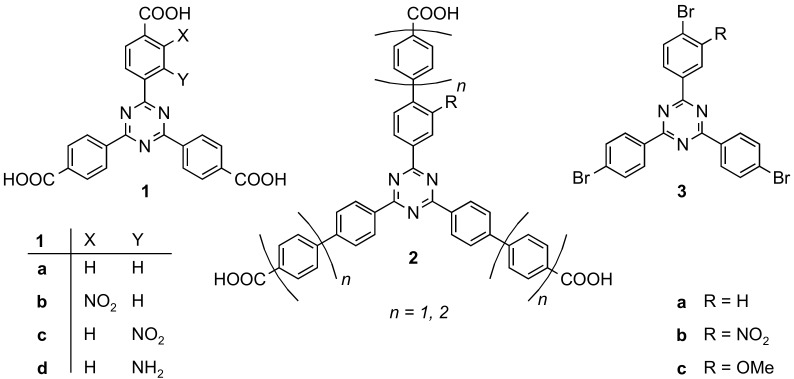
Tridentate mono-substituted triazine based linkers **1** and **2** [13,14]. The elongated tricarboxylic acids **2** were synthesized from the respective tribromides **3a**–**c** using Suzuki-Miyaura couplings.

In this work, we describe the syntheses of mono-functionalized hexadentate ligands. In contrast to the linkers used in NU-109 and NU-110, we have chosen a 1,3,5-triazine as the central aromatic ring because triaryltriazines are more planar than triarylbenzenes [12,13]. A variety of related tridentate and monofunctionalized triazine linkers **1** and **2** have been synthesized (see Figure 1) [14]. Some of them have already been used in the syntheses of MOFs, yielding PCN-6 analogues that contain additional functionalities such as NO_2_ and NH_2_ [13,14,15,16].

## 2. Results and Discussion

**Figure 2:**
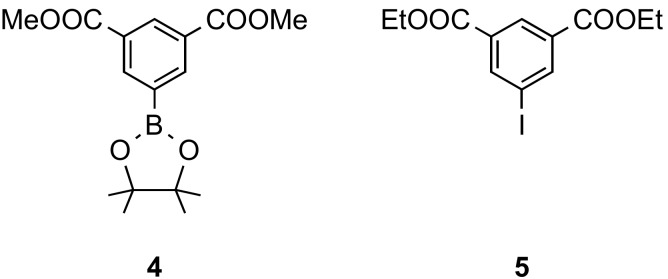
Necessary isophthalates **4** and **5** for the palladium catalyzed cross-coupling reactions to form hexadentate linkers **6** and **8**.

For the extension of **3a**–**c**, Suzuki-Miyaura couplings and other transition metal–catalyzed cross-couplings can be utilized, for instance the Sonogashira-Hagihara reaction. To finally obtain a hexacarboxylate, the coupling partners must contain two carboxylic acid functions. Hence, isophthalic acid derivatives have to be used, many of which are commercially available or have been described in the literature. For the syntheses of the hexadentate linkers **6** and **11**, 5-boron- and 5-iodo-substituted isophthalic derivatives **4** and **5**, respectively, were needed (Figure 2). 

Boronate **4** is commercially available but was synthesized in this work from dimethyl isophthalate via its 5-bromoderivative by palladium(0)-catalyzed boronation with bis(pinacolato)diboron [17]. Anhydrous conditions are necessary to avoid coupling of the brominated starting compound with the product to give an undesired biphenyl derivative carrying four ester groups.

Diethyl iodoisophthalate **5** was synthesized from 5-aminophthalic acid. After esterification, the iodo function was introduced by a Sandmeyer analogous iodination following a procedure from the literature [18].

### 2.1. 2,4,6-Tris(biphenyl)-1,3,5-triazine Hexacarboxylates

**Figure 3:**
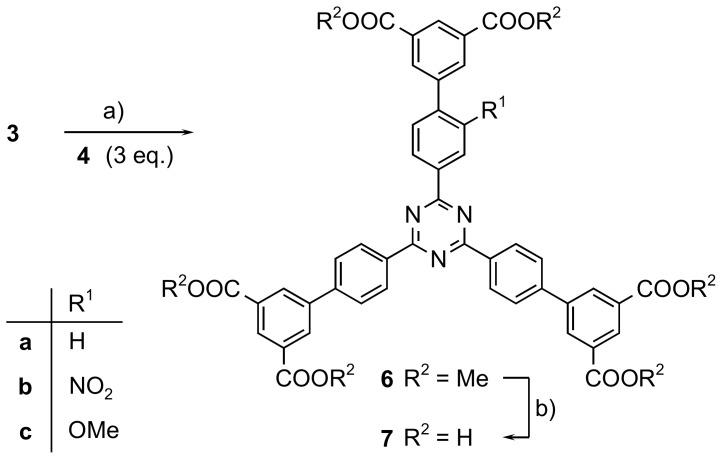
Syntheses of hexadentate mono-substituted triazine based linkers **6** and **7**: a) in dioxane/water (10:1): Pd(dppf)Cl_2_, KOAc (**6a**, 88%), Pd(PPh_3_)_4_, K_3_PO_4_ (**6b**, 83%; **6c**, 81%). b) LiOH, H_2_O (**7a**, quant.; **7b**, quant.; **7c**, 89%).

The Suzuki-Miyaura reaction is a well-established method to connect aromatic rings. Consequently, unsubstituted and nitro- and methoxy-substituted 2,4,6-tribromo-1,3,5-triazines **3a**–**c** were coupled with boronate **4** (Figure 3). The respective hexamethyl hexacarboxylates **6** could be isolated in an 81 to 88% yield. The last step in the synthesis of the functionalized hexadentate linkers **7**, hydrolyses of the esters **6**, was performed with lithium hydroxide in a mixture of water and THF in an 89 to >99% yield. However, it should be noted that the esters can also be used in MOF syntheses, as long as hydrolytic conditions are used. In these cases, the esters are hydrolysed yielding the corresponding carboxylates as the actual linkers.

### 2.2. 2,4,6-Tris(phenylethynylphenyl)-1,3,5-triazine Hexacarboxylates

**Figure 4:**
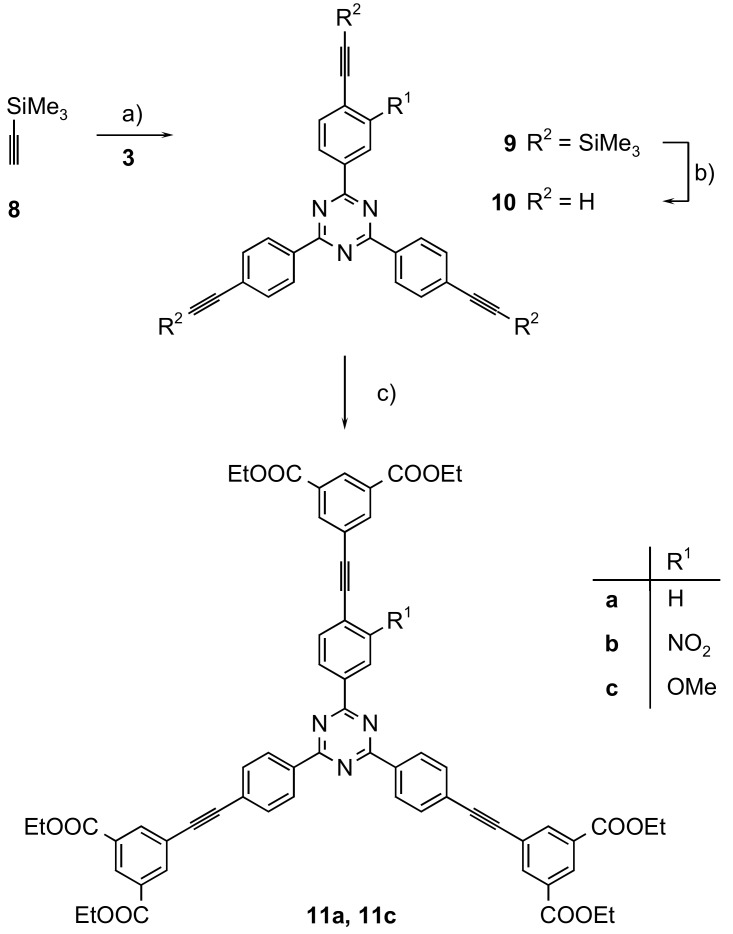
Syntheses of hexadentate mono-substituted alkyne containing triazine based linkers **11**: a) Pd(PPh_3_)_4_, CuI, NEt_3_ (**9a**: 75%; **9b**: 72%; **9c**: 94%); b) K_2_CO_3_, MeOH (**10a**, **10c**: quant.); c) Pd(PPh_3_)_2_Cl_2_, CuI, NEt_3_ (**11a**: 79%; **11c**: 81%).

The reason to use a triazine core for linkers with three arms rather than a benzene ring - i.e. using for instance TATB (1,3,5,-triazine-2,4,6-tribenzoate) instead of BTB (1,3,5-benzenetribenzoate), see above and ref. [12,13] - is the more pronounced planarity of the aromatic rings in the triaryltriazine system. However, in the hexadentate linkers **6** and **7**, biphenyl substructures have been generated by the cross-coupling reaction. The repulsion of the *ortho* hydrogen atoms in the biphenyls will lead to twists in the “arms.” This problem will even be larger if each arm contained more aryl rings—for instance, if it was a *p*-terphenyl. We have therefore chosen an alternative structure by exchanging the central aromatic ring of a potential *p*-terphenyl by an alkyne. NU-109 also contains aryl-alkyne-aryl subunits instead of aryl-aryl ones as present in NU-110 [10]. In terms of coupling chemistry in the syntheses of the linkers, Sonogashira-Hagihara couplings have to be performed instead of Suzuki-Miyaura reactions (Figure 4).

Starting from the tribromides **3a**–**c**, a first triple Sonogashira-Hagihara coupling with trimethylsilyl-protected ethyne **8** gave 2,4,6-tris(trimethylsilylethynylphenyl)-1,3,5-triazines **9** in 72 to 94% yield. The TMS protecting groups in **9** were easily cleaved off by treatment with potassium carbonate in methanol [19]. The unsubstituted and the methoxy-substituted linkers **10a** and **10c** could be isolated in a >99% yield, while the nitro compound **10b** could not be purified sufficiently. Therefore, the final step, a triple Sonogashira reaction of the triynes with diethyl 5-iodoisophthalate **5**, was carried out with **10a** and **c**, and the hexaethyl hexacarboxylates **11a** and **11c** were obtained in yields of 79 and 81%. These yields correspond to >92% yield for each single coupling step.

While the hexaesters **6a**–**c** could be hydrolyzed to yield the corresponding hexaacids **7a**–**c** in analytically pure form (see above), we were not able to isolate the hexaacids derived from **11a** or **c** in a sufficiently pure form. Nevertheless, esters can be employed in MOF syntheses as well because most solvothermal reactions conditions hydrolyze esters anyway. 

## 3. Experimental Section 

General Remarks: 1,1′-Bis(diphenylphosphine)ferrocene-palladium(II) chloride (99.9%, ABCR, Karlsruhe, Germany), bis(triphenylphosphine)palladium(II) dichloride (98%, ABCR), tetrakis(triphenylphosphine)palladium(0) (99%, ABCR), and trimethylsilylethyne (**8**, 98%, ABCR) were purchased and used without further purification. Dry solvents were obtained using suitable desiccants. Other solvents were distilled before use. Melting points were measured with a Gallenkamp MPD350.BM2.5 instrument. NMR spectra were recorded with a Bruker DRX 500 or Avance 600 instrument at 300 K (Billerica, MA, USA). Assignments are supported by COSY, HSQC, and HMBC. Even when obtained by DEPT, the type of ^13^C signal is always listed as singlet, doublet, etc. All chemical shifts are referenced to tetramethylsilane or the residual proton or carbon signals of the solvent. ^1^H and ^13^C-NMR spectra of compounds **6**, **7**, **9**, **10**, and **11** can be found in the Supplementary Materials. HRMS-EI mass spectra were recorded with JEOL AccuTOF GCV 4G (Tokyo, Japan). MALDI-TOF mass spectra were recorded with a Bruker-Daltronics Biflex III (Billerica, MA, USA) with Cl-CCA (4-chloro-α-cyanocinnamic acid) as matrix. IR spectra were recorded with a Perkin-Elmer Spectrum 100 spectrometer (Waltham, MA, USA) equipped with a Golden Gate Diamond ATR unit A-531-G. Elemental analyses were carried out with a Euro EA 3000 Elemental Analyzer from Euro Vector (Pavia, Italy). Traces of solvent originated from the purification step. The analytical sample of **11a** was obtained from an NMR solution.

### 3.1. 2,4,6-Tris[3′,5′-bis(methoxycarbonyl)-biphenyl-4-yl]-1,3,5-triazine *(**6a**)*

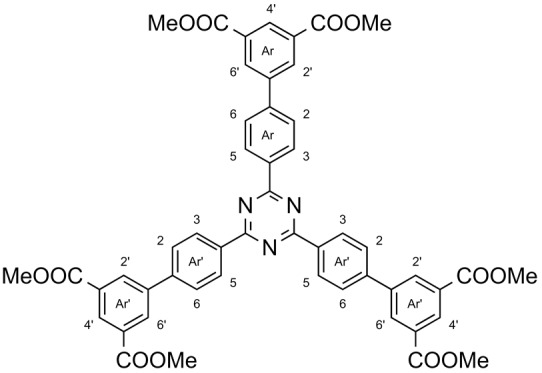



Under nitrogen, a solution of 2,4,6-tris(4-bromophenyl)-1,3,5-triazine (**3a**, 803 mg, 1.47 mmol) [20], dimethyl 5-(4,4,5,5-tetramethyl-1,3,2-dioxaborolan-2-yl)-isophthalate (**4**, 1.69 g, 5.28 mmol), potassium acetate (1.30 g, 13.2 mmol), and 1,1′-bis(diphenylphosphine)ferrocene-palladium(II) chloride (120 mg, 164 µmol) in a 10:1 mixture (90 mL) of 1,4-dioxane and water was heated to reflux for 48 h. After evaporation of the dioxane, deionized water (100 mL) was added, and the aq. layer was extracted with chloroform (3 × 100 mL). The combined organic extract was washed with brine (100 mL), dried with magnesium sulfate, and filtered. Activated charcoal was added, and the mixture was heated and filtered while hot. After reduction of the volume, the crude product was recrystallized from chloroform/petrol ether, yielding 1.14 g (1.29 mmol, 88%) of a colorless solid. M. p.: >300 °C. ^1^H NMR (600 MHz, CDCl_3_): δ = 8.88 (m_c_(d), 6 H, *J* = 8.1 Hz, Ar-*H*-3,5), 8.69 (s, 3 H, Ar-*H*-4′), 8.56 (s, 6 H, Ar-*H*-2′,6′), 7.87 (m_c_(d), 6 H, *J* = 8.1 Hz, Ar-*H*-2,6), 4.01 (s, 18 H, CO_2_C*H*_3_) ppm. ^13^C NMR (150 MHz, CDCl_3_): δ = 171.2 (s, tri-*C*-2,4,6), 166.1 (s, *C*O_2_Me), 142.9 (s, Ar-*C*-1), 141.1 (s, Ar-*C*-1′), 136.0 (s, Ar-*C*-4), 132.4 (d, Ar-*C*-2′,6′), 131.3 (s, Ar-*C*-3′,5′), 129.9 (d, Ar-*C*-4′), 129.7 (d, Ar-*C*-3,5), 127.4 (d, Ar-*C*-2,6), 52.6 (q, CO_2_*C*H_3_) ppm. MS (MALDI, Cl-CCA): *m*/*z* = 886 [M + H]^+^. IR (ATR): $\tilde{\nu }$ = 3005 (aryl-H), 2993 (C-H-val.), 1690 (C=O), 1609, 1581, 1509 (arom. C=C, arom. C=N), 1435 (CH-def.), 1349 (C-N-val.), 813 (1,4-disubst. aryl, 1,3,5-trisubst. aryl) cm^−1^. Elemental analysis (C_51_H_39_N_3_O_12_) (885.87): calcd. C 69.15 H 4.44 N 4.74; (C_51_H_39_N_3_O_12_·0.1 CHCl_3_) (897.81): calcd. C 68.36 H 4.39 N 4.68; found C 68.26 H 4.28 N 4.81.

### 3.2. 2-[3′,5′-Bis(methoxycarbonyl)-2-nitrobiphenyl-4-yl]-4,6-bis[3′,5′-bis(methoxycarbonyl)-biphenyl-4-yl]-1,3,5-triazine *(**6b**)*

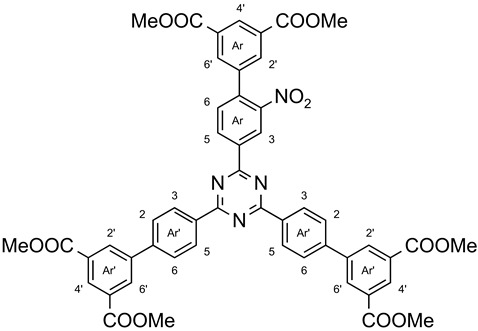



Under nitrogen, a mixture of 2-(4-bromo-3-nitrophenyl)-4,6-bis(4-bromophenyl)-1,3,5-triazine (**3b**, 100 mg, 170 µmol) [13], dimethyl 5-(4,4,5,5-tetramethyl-1,3,2-dioxaborolan-2-yl)-isophthalate (**4**, 245 mg, 765 µmol), tetrakis(triphenylphosphine)-palladium(0) (30 mg, 26 µmol), and potassium phosphate (234 mg, 1.10 mmol) in a mixture of 1,4-dioxane (10 mL) and deionized water (1 mL) was heated to reflux for 48 h. After evaporation of the dioxane in vacuo, the residue was dissolved in water (25 mL) and extracted with chloroform (3 × 25 mL). The combined organic layer was washed with brine (25 mL), dried with magnesium sulfate, and filtered. Activated charcoal was added to the filtrate, and the mixture was heated and filtered through celite while hot. The solvent was evaporated in vacuo and the residue was recrystallized from a hot mixture of toluene and *n*-heptane yielding 131 mg (141 µmol, 83%) of a colorless solid, m. p.: >300 °C. ^1^H NMR (500 MHz, CDCl_3_): δ = 9.33 (s, 1 H, Ar-*H*-3), 9.02 (d, 1 H, *J* = 7.0 Hz, Ar-*H*-5), 8.86 (d, 4 H, *J* = 8.0 Hz, Ar′-*H*-3,5), 8.75 (s, 1 H, Ar-*H*-4′), 8.69 (s, 2 *H*, Ar-*H*-2′,6′), 8.53 (s, 4 H, Ar′-*H*-2′,6′), 8.23 (s, 2 H, Ar′-*H*-4′), 7.87 (d, 4 H, *J* = 8.0 Hz, Ar′-*H*-2,6), 7.65 (d, 1 H, *J* = 7.0 Hz, Ar-*H*-6), 4.01 (s, 12 H, Ar′-CO_2_C*H*_3_), 3.98 (s, 6 H, Ar-CO_2_C*H*_3_) ppm. ^13^C NMR (125 MHz, CDCl_3_): δ = 171.6 (s, tri-*C*-4,6), 169.3 (s, tri-*C*-2), 166.0 (s, Ar-*C*O_2_Me), 166.0 (s, Ar′-*C*O_2_Me), 149.1 (s, Ar-*C*-2), 143.3 (s, Ar′-*C*-1), 140.8 (s, Ar′-*C*-1′), 138.0 (s, Ar-*C*-1), 137.2 (Ar-*C*-1′), 135.2 (s, Ar′-*C*-4), 133.1 (d, Ar′-*C*-4′), 132.5 (d, Ar-*C*-5), 132.4 (d, Ar′-*C*-2′,6′), 132.4 (d, Ar-*C*-6), 131.4 (s, Ar′-*C*-3′,5′), 131.3 (s, Ar-*C*-3′,5′), 130.6 (d, Ar-*C*-4′), 130.0 (s, Ar-*C*-4), 129.9 (d, Ar-*C*-2′,6′), 129.7 (d, Ar′-*C*-3,5), 127.5 (d, Ar′-*C*-2,6), 124.8 (d, Ar-*C*-3), 52.6 (q, Ar-CO_2_*C*H_3_), 52.6 (q, Ar′-CO_2_*C*H_3_) ppm. MS (MALDI, Cl-CCA): *m*/*z* = 931 [M + H]^+^. IR (ATR): $\tilde{\nu }$ = 3005 (aryl-H), 2992, 2952 (C-H-val.), 1719 (C=O), 1607, 1575, 1506 (arom. C=C, arom. C=N), 1515 (NO_2_), 1431 (CH-def.), 1341 (C-N-val.), 817 (1,4-disubst. aryl, 1,3,4-trisubst. aryl) cm^−1^. Elemental analysis (C_51_H_38_N_4_O_14_) (930.87): calcd. C 65.80 H 4.11 N 6.02; found C 65.50 H 4.12 N 5.91.

### 3.3. 2-[2-Methoxy-3′,5′-bis(methoxycarbonyl)-biphenyl-4-yl]-4,6-bis[3′,5′-bis(methoxycarbonyl)-biphenyl-4-yl]-1,3,5-triazine *(**6c**)*

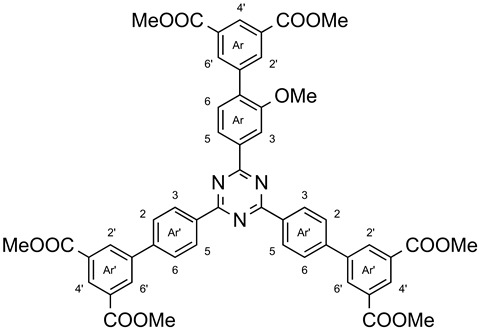



Under nitrogen, 2-(4-bromo-3-methoxyphenyl)-4,6-bis(4-bromophenyl)-1,3,5-triazine (**3c**, 100 mg, 175 µmol) [13], dimethyl 5-(4,4,5,5-tetramethyl-1,3,2-dioxaborolan-2-yl)-isophthalate (**4**, 252 mg, 788 µmol) and potassium phosphate (234 mg, 1.10 mmol) were mixed with a mixture of 1,4-dioxane (10 mL), and deionized water (1 mL). Tetrakis(triphenylphosphine)-palladium(0) (30 mg, 26 µmol) was added, and the mixture was stirred for 48 h at 100 °C. After evaporation of the dioxane in vacuo, chloroform (25 mL) was added to the residue, and the resulting mixture was washed with deionized water (3 × 25 mL). The organic layer was dried with magnesium sulfate, filtered, and heated with little activated charcoal. After filtration through celite while hot, the solvent was evaporated in vacuo until the residue turned turbide. Recrystallization from a boiling mixture of chloroform and petrol ether (b. p. 40–60 °C) yielded 130 mg (142 µmol, 81 %) of a colorless solid, m. p.: >300 °C. ^1^H NMR (500 MHz, CDCl_3_): δ = 8.88 (d, 4 H, *J* = 8.5 Hz, Ar′-*H*-3,5), 8.70 (t, 2 H, *J* = 1.5 Hz, Ar′-*H*-4′), 8.67 (t, 1 H, *J* = 1.6 Hz, Ar-*H*-4′), 8.55 (d, 4 H, *J* = 1.5 Hz, Ar′-*H*-2′,6′), 8.49 (dd, 1 H, *J* = 7.9 Hz, *J* = 1.4 Hz, Ar-*H*-5), 8.47 (d, 2 H, *J* = 1.6 Hz, Ar-*H*-2′,6′), 8.42 (d, 1 H, *J* = 1.4 Hz, Ar-*H*-3), 7.88 (d, 4 H, *J* = 8.5 Hz, Ar′-*H*-2,6), 7.56 (d, 1 H, *J* = 7.9 Hz, Ar-*H*-6), 4.07 (s, 3 H, Ar-OC*H*_3_), 4.01 (s, 12 H, Ar′-CO_2_C*H*_3_), 3.99 (s, 6 H, Ar-CO_2_C*H*_3_) ppm. ^13^C NMR (125 MHz, CDCl_3_): δ = 171.2 (s, tri-*C*-4,6), 171.1 (s, tri-*C*-2), 166.3 (s, Ar-*C*O_2_Me), 166.1 (s, Ar′-*C*O_2_Me), 156.7 (s, Ar-*C*-2), 143.0 (s, Ar′-*C*-1), 141.0 (s, Ar′-*C*-1′), 138.7 (s, Ar-*C*-1′), 137.4 (s, Ar-*C*-4), 135.9 (s, Ar′-*C*-4), 134.9 (d, Ar-*C*-2′,6′), 132.7 (s, Ar-*C*-1), 132.3 (d, Ar′-*C*-2′,6′), 131.4 (s, Ar-*C*-3′,5′). 131.0 (d, Ar-*C*-6), 130.5 (s, Ar′-*C*-3′,5′), 129.9 (d, Ar′-*C*-4′), 129.7 (d, Ar′-*C*-3,5), 129.6 (d, Ar-*C*-4′), 127.4 (d, Ar′-*C*-2,6), 121.9 (d, Ar-*C*-5), 111.2 (d, Ar-*C*-3), 55.9 (q, Ar-O*C*H_3_), 52.5 (q, Ar′-CO_2_*C*H_3_), 52.4 (q, Ar-CO_2_*C*H_3_) ppm. MS (MALDI, Cl-CCA): *m*/*z* = 916 [M + H]^+^. IR (ATR): $\tilde{\nu }$^~^ = 3005 (aryl-H), 2954 (C-H-val.), 1728 (C=O), 1606, 1578, 1517 (arom. C=C, arom. C=N), 1429 (CH-def.), 1371 (OCH_3_), 1342 (C-N-val.), 809 (1,4-disubst. aryl, 1,3,4-trisubst. aryl) cm^−1^. Elemental analysis (C_52_H_41_N_3_O_13_) (915.89): calcd. C 68.19 H 4.51 N 4.59; (C_52_H_41_N_3_O_13_·0.2 CHCl_3_) (939.71): calcd. C 66.71 H 4.42 N 4.47; found C 66.34 H 4.74 N 4.86.

### 3.4. 2,4,6-Tris[3′,5′-dicarboxybiphenyl-4-yl]-1,3,5-triazine *(**7a**)*

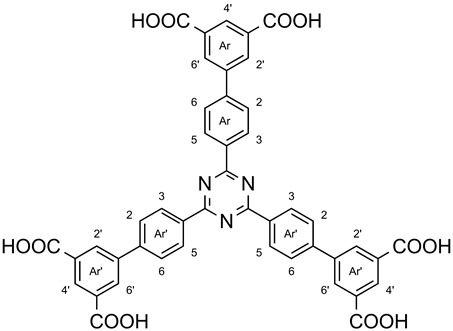



A mixture of 2,4,6-tris[3′,5′-bis(methoxycarbonyl)-biphenyl-4-yl]-1,3,5-triazine (**6a**, 1.04 g, 1.17 mmol) and lithium hydroxide monohydrate (1.38 g, 32.8 mmol) in tetrahydrofuran (130 mL) and deionized water (15 mL) was heated to 60 °C for 48 h. After evaporation of the solvent in vacuo, a small amount of deionized water was added, and the mixture was acidified with hydrochloric acid (6 M). The precipitate was filtered off, washed with deionized water and chloroform, and dried in vacuo, yielding 939 mg (1.17 mmol, > 99%) of a yellow solid, m. p.: >300 °C. ^1^H NMR (600 MHz, DMSO-*d*_6_): δ = 8.76 (d, 6 H, *J* = 8.3 Hz, Ar-*H*-3,5), 8.47 (t, 3 H, *J* = 1.4 Hz, Ar-*H*-4′), 8.41 (d, 6 H, *J* = 1.4 Hz, Ar-*H*-2′,6′), 7.94 (d, 6 H, *J* = 8.3 Hz, Ar-*H*-2,6) ppm. ^13^C NMR (150 MHz, DMSO-d6): δ = 170.6 (s, tri-*C*-2,4,6), 166.4 (s, *C*O_2_H), 142.5 (s, Ar-*C*-1), 140.0 (s, Ar-*C*-1′), 135.0 (s, Ar-*C*-4), 132.2 (s, Ar-*C*-3′,5′), 131.4 (d, Ar-*C*-2′,6′), 129.5 (d, Ar-*C*-4′), 128.8 (d, Ar-*C*-3,5), 127.4 (d, Ar-*C*-2,6) ppm. MS (MALDI, Cl-CCA): *m*/*z* = 802 [M + H]^+^. IR (ATR): $\tilde{\nu }$ = 3018 (br., OH), 1690 (C=O), 1603, 1578, 1508, 1412 (arom. C=C, arom. C=N), 1367 (C-N-val.), 815 (1,3,5-trisubst. aryl) cm^−1^. Elemental analysis (C_45_H_27_N_3_O_12_) (801.71): calcd. C 67.42 H 3.39 N 4.87; (C_45_H_27_N_3_O_12_·1.4 H_2_O·0.2 CHCl_3_) (850.80): calcd. C 63.81 H 3.55 N 4.94; found C 63.87 H 3.67 N 4.87.

### 3.5. 2-[3′,5′-Dicarboxy-2-nitrobiphenyl-4-yl]-4,6-bis[3′,5′-dicarboxybiphenyl-4-yl]-1,3,5-triazine *(**7b**)*

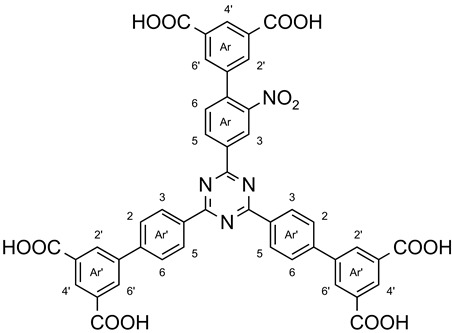



A suspension of 2-[3′,5′-bis(methoxycarbonyl)-2-nitrobiphenyl-4-yl]-4,6-bis[3′,5′-bis(methoxycarbonyl)-biphenyl-4-yl]-1,3,5-triazine (**6b**, 44 mg, 47 µmol) and lithium hydroxide monohydrate (101 mg, 2.41 mmol) in a mixture of tetrahydrofuran (10 mL) and deionized water (1.5 mL) was stirred at 60 °C for 24 h. After evaporation of the tetrahydrofuran in vacuo, some deionized water was added to the residue. Acidification with hydrochloric acid (6 M) produced a precipitate which was filtered off and washed thoroughly with deionized water and chloroform yielding 39 mg (46 µmol, >99%) of a yellow solid, m. p.: >300 °C. ^1^H NMR (500 MHz, DMSO-d6): δ = 9.21 (d, 1 H, *J* = 1.6 Hz, Ar-*H*-3), 9.01 (dd, 1 H, *J* = 8.0 Hz, *J* = 1.6 Hz, Ar-*H*-5), 8.81 (d, 4 H, *J* = 8.4 Hz, Ar′-*H*-3,5), 8.53 (t, 1 H, *J* = 1.5 Hz, Ar-*H*-4′), 8.48 (t, 2 H, *J* = 1.5 Hz, Ar′-*H*-4′), 8.43 (d, 4 H, *J* = 1.5 Hz, Ar′-*H*-2′,6′), 8.15 (d, 2 H, *J* = 1.5 Hz, Ar-*H*-2′,6′), 7.98 (d, 4 H, *J* = 8.4 Hz, Ar′-*H*-2,6), 7.88 (d, 1 H, *J* = 8.0 Hz, Ar-*H*-6) ppm. ^13^C NMR (125 MHz, DMSO-d6): δ = 171.4 (s, tri-*C*-4,6), 169.4 (s, tri-*C*-2), 166.8 (s, Ar′-*C*O_2_H), 166.5 (s, Ar-*C*O_2_H), 149.3 (s, Ar-*C*-2), 143.3 (s, Ar′-*C*-1), 140.9 (s, Ar-*C*-4), 140.4 (s, Ar′-*C*-4), 137.7 (s, Ar-*C*-1), 137.1 (s, Ar-*C*-4), 136.8 (s, Ar-*C*-1′), 135.1 (s, Ar′-*C*-1′), 133.4 (d, Ar-*C*-6), 133.0 (d, Ar-*C*-5), 132.9 (d, Ar-*C*-2′,6′), 132.6 (s, Ar-*C*-3′,5′), 132.4 (s, Ar′-*C*-3′,5′), 131.9 (d, Ar′-*C*-2′,6′), 130.2 (d, Ar-*C*-4′), 130.1 (d, Ar′-*C*-3,5), 129.99 (s, Ar-*C*-1′), 129.95 (Ar′-*C*-4′), 127.9 (d, Ar′-*C*-2,6), 124.5 (d, Ar-*C*-3) ppm. MS (MALDI, Cl-CCA): *m*/*z* = 847 [M + H]^+^. IR (ATR): $\tilde{\nu }$ = 3080 (OH), 2921, 2854 (CO_2_H), 1696 (C=O), 1608, 1576, 1515, 1456 (arom. C=C, arom. C=N), 1360 (C-N-val.), 1243 (NO_2_), 813 (1,4-disubst. aryl, 1,3,5-trisubst. aryl) cm^−1^. Elemental analysis (C_45_H_26_N_4_O_14_) (846.71): calcd. C 63.83 H 3.10 N 6.62; (C_45_H_26_N_4_O_14_·0.95 CHCl_3_·0.05 H_2_O) (960.95): calcd. C 57.43 H 2.84 N 5.83; found C 57.80 H 3.04 N 5.43.

### 3.6. 2,4-Bis[3′,5′-dicarboxybiphenyl-4-yl]-6-[3′,5′-dicarboxy-2-methoxybiphenyl-4-yl]-1,3,5-triazine *(**7c**)*

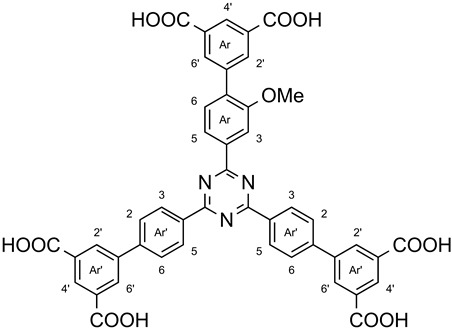



A suspension of 2-[2-methoxy-3′,5′-bis(methoxycarbonyl)-biphenyl-4-yl]-4,6-bis[3′,5′-bis(methoxycarbonyl)-biphenyl-4-yl]-1,3,5-triazine (**6c**, 50 mg, 54 µmol) and lithium hydroxide monohydrate (101 mg, 2.41 mmol) in a mixture of tetrahydrofuran (10 mL) and deionized water (1.5 mL) was heated to 60 °C for 24 h. After evaporation of tetrahydrofuran in vacuo, the aqueous residue was diluted slightly with deionized water and acidified with hydrochloric acid (6 M). The precipitate was washed with deionized water and chloroform, yielding 40 mg (48 µmol, 89%) of a yellow solid, m. p.: >300 °C. ^1^H NMR (600 MHz, DMSO-d6): δ = 13.39 (br. s, 6 H, CO_2_*H*), 8.84 (d, 4 H, *J* = 8.2 Hz, Ar′-*H*-3,5), 8.51–8.50 (m, 2 H, Ar′-*H*-4′), 8.48–8.47 (m, 5 H, Ar′-*H*-2′,6′, Ar-*H*-4′), 8.44 (m_c_(d), 1 H, *J* = 7.8 Hz, Ar-*H*-5), 8.41 (s, 1 H, Ar-*H*-3), 8.34 (m_c_(d), 2 H, *J* = 1.1 Hz, Ar-*H*-2′,6′), 8.01 (d, 4 H, *J* = 8.2 Hz, Ar′-*H*-2,6), 7.66 (d, 1 H, *J* = 7.8 Hz, Ar-*H*-6), 4.04 (s, 3 H, OC*H*_3_) ppm. ^13^C NMR (150 MHz, DMSO-d6): δ = 170.7(s, tri-*C*-4,6), 170.4 (s, tri-*C*-2), 166.5 (s, Ar-*C*O_2_*H*), 166.4 (s, Ar′-*C*O_2_*H*), 156.4 (s, Ar-*C*-2), 142.6 (s, Ar′-*C*-1), 140.0 (s, Ar′-*C*-1′), 137.9 (s, Ar-*C*-1′), 136.7 (s, Ar-*C*-4), 135.1 (s, Ar′-*C*-4), 134.0 (d, Ar-*C*-2′,6′), 132.3 (s, Ar-*C*-1), 132.2 (s, Ar′-*C*-3′,5′), 131.5 (d, Ar-*C*-5), 131.3 (s, Ar-*C*-3′,5′), 131.0 (d, Ar-*C*-6), 129.6 (d, Ar′-*C*-3,5), 129.3 (d, Ar′-*C*-2′,6′), 129.0 (d, Ar′-*C*-4′), 127.5 (d, Ar′-*C*-2,6), 121.6 (d, Ar-*C*-4′), 111.2 (d, Ar-*C*-3), 55.8 (q, O*C*H_3_) ppm. MS (MALDI, Cl-CCA): *m*/*z* = 832 [M + H]^+^. IR (ATR): $\tilde{\nu }$ = 3100 (br., OH), 1692 (C=O), 1603, 1578, 1509, 1405 (arom. C=C, arom. C=N), 1360 (C-N-val.), 1221 (aryl-OCH_3_), 810 (1,3,5-trisubst. aryl) cm^−1^. Elemental analysis (C_46_H_29_N_3_O_13_) (831.17): calcd. C 67.42 H 3.39 N 4.87; (C_46_H_29_N_3_O_13_·0.45 H_2_O·0.9 CHCl_3_) (947.28): calcd. C 59.47 H 3.28 N 4.44; found C 59.58 H 3.40 N 4.48.

### 3.7. 2,4,6-Tris{4-[(trimethylsilyl)ethynyl]-phenyl}-1,3,5-triazine *(**9a**)*

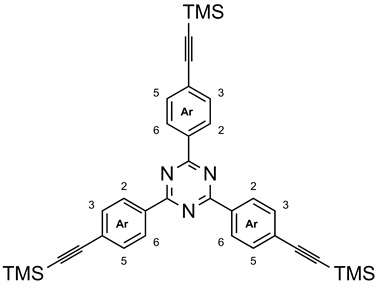



Under nitrogen, trimethylsilylethyne (**8**, 972 μL, 6.88 mmol) was added to a mixture of 2,4,6-tris(4-bromophenyl)-1,3,5-triazine (***3a***, 1.00 g, 1.83 mmol) [20] in tetrahydrofuran (200 mL) and triethylamine (120 mL). After the addition of tetrakis(triphenylphosphine)-palladium(0) (240 mg, 208 µmol), copper(I) iodide (40 mg, 208 µmol), and triethylamine (120 mL), the mixture was stirred for 48 h at 40 °C (TLC control, silica gel, cyclohexane, *R*_f_ = 0.23). Solvents were distilled off in vacuo, and the residue was mixed with deionized water (100 mL) and extracted with ethyl acetate (3 × 100 mL). The combined organic layer was washed with brine (100 mL), dried with magnesium sulfate, and filtered. The solvent was distilled off in vacuo, and the residue was dissolved in toluene and filtered through neutral aluminium oxide. After evaporation of the solvent in vacuo, the residue was recrystallized from boiling *n*-heptane yielding 826 mg (1.38 mmol, 75%) of a colorless solid, m. p.: 274 °C (ref. [19]: no m.p. given). ^1^H NMR (600 MHz, CDCl_3_): δ = 8.67 (m_c_(d), 6 H, *J* = 8.4 Hz, Ar-*H*-3,5), 7.64 (m_c_(d), 6 H, *J* = 8.4 Hz, Ar-*H*-2,6), 0.30 (s, 27 H, Si(C*H*_3_)_3_) ppm. ^13^C NMR (150 MHz, CDCl_3_): δ = 171.0 (s, tri-*C*-2,4,6), 135.7 (s, Ar-*C*-1), 132.2 (d, Ar-*C*-2,6), 128.7 (d, Ar-*C*-3,5), 127.4 (s, Ar-*C*-4), 104.7 (s, Ar-*C*≡C), 97.5 (s, Ar-C≡*C*), 0.0 (s, Si(*C*H_3_)_3_) ppm. HRMS (EI): *m*/*z* = calcd. 597.2451; found 597.2441 (Δ 1.78 ppm). Elemental analysis (C_36_H_39_N_3_Si_3_) (597.97): calcd. C 72.31 H 6.57 N 7.03; found C 72.17 H 6.52 N 6.93.

### 3.8. 2-{3-Nitro-4-[(trimethylsilyl)ethynyl]-phenyl}-4,6-bis{4-[(trimethylsilyl)ethynyl]-phenyl}-1,3,5-triazine *(**9b**)*

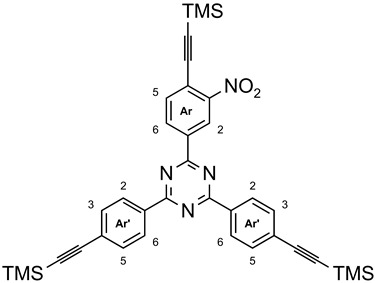



Under nitrogen, tetrakis(triphenylphosphine)-palladium(0) (88 mg, 77 µmol) and copper(I) iodide (15 mg, 77 µmol) were added to a mixture of 2-(4-bromo-3-nitrophenyl)-4,6-bis(4-bromophenyl)-1,3,5-triazine (**3b**, 500 mg, 850 µmol) [13] in tetrahydrofuran (100 mL) and triethylamine (60 mL). After the addition of trimethylsilylethyne (**8**, 726 µL, 5.10 mmol), the mixture was stirred for 48 h at 55 °C (TLC control, silica gel, cyclohexane/ethyl acetate, 10:1, *R*_f_ = 0.76). The solvent was evaporated in vacuo, and chloroform (150 mL) was added to the residue. The organic layer was washed with deionized water (3 × 100 mL), dried with magnesium sulfate, and filtered. Activated charcoal was added, and the mixture was heated and filtered through celite and silica gel. The solvent was evaporated in vacuo, and the crude product was recrystallized from boiling *n*-heptane yielding 391 mg (608 µmol, 72%) of a colorless solid, m. p.: 273–275 °C. ^1^H NMR (500 MHz, CDCl_3_): δ = 9.31 (d, 1 H, *J* = 1.4 Hz, Ar-*H*-2), 8.88 (dd, 1 H, *J* = 8.1 Hz, *J* = 1.4 Hz, Ar-*H*-6), 8.67 (d, 4 H, *J* = 8.4 Hz, Ar′-*H*-2,6), 7.82 (d, 1 H, *J* = 8.1 Hz, Ar-*H*-5), 7.65 (d, 4 H, *J* = 8.4 Hz, Ar′-*H*-3,5), 0.32 (s, 9 H, Ar-C≡C-Si(C*H*_3_)_3_), 0.30 (s, 18 H, Ar′-C≡C-Si(C*H*_3_)_3_) ppm. ^13^C NMR (125 MHz, CDCl_3_): δ = 171.4 (s, tri-*C*-4,6), 169.2 (s, tri-*C*-2), 150.7 (s, Ar-*C*-3), 136.9 (s, Ar-*C*-1), 135.4 (d, Ar-*C*-5), 135.2 (s, Ar′-*C*-1), 132.3 (d, Ar′-*C*-3,5), 132.2 (d, Ar-*C*-6), 128.8 (d, Ar-*C*-2,6), 127.9 (s, Ar′-*C*-4), 124.7 (d, Ar-*C*-2), 121.7 (s, Ar-*C*-4), 107.2 (s, Ar-*C*≡C), 104.5 (s, Ar-C≡*C*), 99.2 (s, Ar′-*C*≡C), 98.0 (s, Ar′-C≡*C*), 0.3 (q, Ar′-C≡C-Si(*C*H_3_)_3_), 0.0 (q, Ar-C≡C-Si(*C*H_3_)_3_) ppm. IR (ATR): $\tilde{\nu }$ = 3005 (aryl-H), 2957, 2901 (C-H-val.), 2155 (C≡C), 1603, 1570, 1504, 1407 (arom. C=C, arom. C=N), 1537 (NO_2_), 1355 (C-N-val.), 838 (1,4-disubst. aryl, 1,3,4-trisubst. aryl) cm^−1^. HRMS (EI): *m*/*z* = calcd. 642.2302; found 642.2295 (Δ 1.11 ppm). Elemental analysis (C_36_H_38_N_4_O_2_Si_3_) (642.97): calcd. C 67.25 H 5.96 N 8.71; found C 67.32 H 6.25 N 8.46.

### 3.9. 2-{3-Methoxy-4-[(trimethylsilyl)ethynyl]-phenyl}-4,6-bis{4-[(trimethylsilyl)ethynyl]-phenyl}-1,3,5-triazine *(**9c**)*

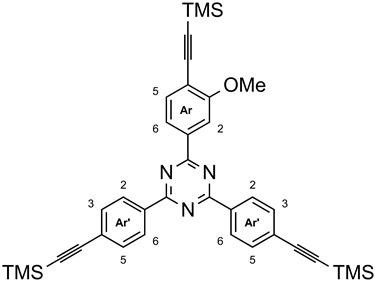



Under nitrogen, tetrakis(triphenylphosphine)-palladium(0) (45 mg, 39 µmol) and copper(I) iodide (8 mg, 0.04 mmol) were added to a mixture of 2-(4-bromo-3-methoxyphenyl)-4,6-bis(4-bromophenyl)-1,3,5-triazine (**3c**, 250 mg, 434 µmol) [13] in tetrahydrofuran (50 mL) and triethylamine (30 mL). Trimethylsilylethyne (**8**, 370 µL, 2.60 mmol) was added and the mixture was stirred for 48 h at 55 °C. After removal of the volatiles in vacuo, the residue was dissolved in chloroform (50 mL) and washed with deionized water (3 × 50 mL). The organic layer was dried with magnesium sulfate, filtered, and heated with activated charcoal. After filtration through celite, the solvent was removed in vacuo. The residue was recrystallized from boiling *n*-heptane yielding 255 mg (406 µmol, 94%) of a colorless solid, m. p. 212 °C. ^1^H NMR (500 MHz, CDCl_3_): δ = 8.67 (m_c_(d), 4 H, *J* = 8.7 Hz, Ar′-*H*-2,6), 8.31 (dd, 1 H, *J* = 8.0 Hz, *J* = 1.4 Hz, Ar-*H*-6), 8.24 (d, 1 H, *J* = 1.4 Hz, Ar-*H*-2), 7.65 (m_c_(d), 4 H, *J* = 8.7 Hz, Ar′-*H*-3,5), 7.61 (d, 1 H, *J* = 8.0 Hz, Ar-*H*-5), 4.08 (s, 3 H, OC*H*_3_), 0.31 (s, 9 H, Ar-C≡C-Si(C*H*_3_)_3_), 0.29 (s, 18 H, Ar′-C≡C-Si(C*H*_3_)_3_) ppm. ^13^C NMR (125 MHz, CDCl_3_): δ = 171.0 (s, tri-*C*-4,6), 171.0 (s, tri-*C*-2), 160.6 (s, Ar-*C*-3), 137.4 (s, Ar-*C*-1), 135.7 (s, Ar′-*C*-1), 134.3 (d, Ar-*C*-2), 132.3 (d, Ar′-*C*-3,5), 128.7 (d, Ar′-*C*-2,6), 127.5 (s, Ar′-*C*-4), 121.2 (d, Ar-*C*-6), 116.8 (s, Ar-*C*-4), 110.5 (d, Ar-*C*-5), 104.6 (s, Ar-*C*≡C), 101.8 (s, Ar-C≡*C*), 100.9 (s, Ar′-*C*≡C), 97.6 (s, Ar′-C≡*C*), 56.2 (q, O*C*H_3_), 0.00 (s, Ar-C≡C-Si(*C*H_3_)_3_), 0.01 (q, Ar′-C≡C-Si(*C*H_3_)_3_) ppm. IR (ATR): $\tilde{\nu }$ = 3005 (aryl-H), 2972 (C-H-val.), 2903 (OCH_3_), 2060 (C≡C), 1606. 1570, 1511, 1407 (arom. C=C, arom. C=N), 1357 (C-N-val.), 813 (1,4-disubst. aryl, 1,3,4-trisubst. aryl) cm^−1^. HRMS (EI): *m*/*z* = calcd. 672.2557; found 672.2543 (Δ 2.38 ppm). Elemental analysis (C_37_H_41_N_3_OSi_3_) (628.00): calcd. C 70.76 H 6.58 N 6.69; (C_37_H_41_N_3_OSi_3_·0.3 C_7_H_16_·0.4 H_2_O) (664.93): calcd. C 70.63 H 7.06 N 6.32; found C 70.97 H 6.78 N 5.99.

### 3.10. 2,4,6-Tris(4-ethynylphenyl)-1,3,5-triazine *(**10a**)*

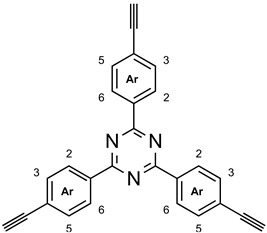



A mixture of 2,4,6-tris{4-[(trimethylsilyl)ethynyl]-phenyl}-1,3,5-triazine (**9a**, 500 mg, 835 µmol) and potassium carbonate (1.04 g, 7.50 mmol) in methanol (25 mL) was stirred for 24 h at room temp. (TLC control, silica gel, cyclohexane, *R*_f_ = 0.15). After evaporation of the methanol, the residue was dissolved in deionized water (25 mL) and extracted with chloroform (3 × 25 mL). The combined organic layer was washed with brine (25 mL) and dried with magnesium sulfate. After filtration and removal of the solvent in vacuo, the crude product was recrystallized from a boiling mixture of toluene and *n*-heptane, yielding 315 mg (827 µmol, >99%) of a yellowish solid, m. p. >300 °C (ref. [19]: no m.p. given). ^1^H NMR (500 MHz, CDCl_3_): δ = 8.63 (m_c_(d), 6 H, *J* = 8.9 Hz, Ar-*H*-3,5), 7.61 (m_c_(d), 6 H, *J* = 8.9 Hz, Ar-*H*-2,6), 3.24 (s, 3 H, C≡CH) ppm. ^13^C NMR (125 MHz, CDCl_3_): δ = 170.9 (s, tri-*C*-2,4,6), 135.9 (s, Ar-*C*-1), 132.2 (d, Ar-*C*-2,6), 128.6 (d, Ar-*C*-3,5), 126.3 (s, Ar-*C*-4), 83.10 (s, *C*≡CH), 80.0 (d, C≡*C*H) ppm. IR (ATR): $\tilde{\nu }$ = 3236 (C≡C-H), 3002 (aryl-H), 2160 (C≡C), 1606, 1574, 1505, 1408 (arom. C=C, arom. C=N), 1357 (C-N-val.), 813 (1,4-disubst. aryl) cm^−1^. HRMS (EI): *m/z* = calcd. 381.1266; found 381.1251 (Δ 3.85 ppm). Elemental analysis (C_27_H_15_N_3_) (381.43): calcd. C 85.02 H 3.96 N 11.02; found C 85.24 H 3.94 N 10.64.

### 3.11. 2,4-Bis(4-ethynylphenyl)-6-(3-methoxy-4-ethynylphenyl)-1,3,5-triazine *(**10c**)*

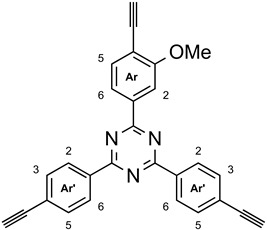



A mixture of 2-{3-methoxy-4-[(trimethylsilyl)ethynyl]-phenyl}-4,6-bis{4-[(trimethylsilyl)ethynyl]-phenyl}-1,3,5-triazine (**9c**, 150 mg, 239 µmol) and potassium carbonate (297 mg, 2.15 mmol) in methanol (7.5 mL) was stirred for 24 h at room temp. After evaporation of the solvent in vacuo, the residue was dissolved in chloroform (25 mL) and washed with water (3 × 25 mL). The organic layer was dried with magnesium sulfate, filtered, and the solvent was evaporated in vacuo. The crude product was recrystallized from a boiling mixture of toluene and *n*-heptane, yielding 98 mg (239 µmol, >99%) of **10c**, m. p. >300 °C. ^1^H NMR (600 MHz, CDCl_3_): δ = 8.71 (d, 4 H, *J* = 8.3 Hz, Ar′-*H*-2,6), 8.35 (dd, 1 H, *J* = 7.8 Hz, *J* = 1.2 Hz, Ar-*H*-6), 8.29 (br. s, 1 H, Ar-*H*-2), 7.70 (d, 4 H, *J* = 8.3 Hz, Ar′-*H*-3,5), 7.66 (d, 1 H, *J* = 7.8 Hz, Ar-*H*-5), 4.12 (s, 3 H, OC*H*_3_), 3.50 (s, 1 H, Ar-C≡C*H*), 3.28 (s, 1 H, Ar′-C≡C*H*) ppm. ^13^C NMR (150 MHz, CDCl_3_): δ = 171.1 (s, tri-*C*-2), 171.1 (tri-*C*-4,6), 160.5 (s, Ar-*C*-3), 136.1 (s, Ar′-*C*-1), 134.3 (d, Ar-*C*-5), 132.4 (d, Ar′-*C*-3,5), 131.9 (s, Ar-*C*-1), 128.8 (d, Ar′-*C*-2,6), 126.4 (s, Ar′-*C*-4), 121.2 (d, Ar-*C*-6), 115.5 (s, Ar-*C*-4), 83.7 (d, Ar-C≡*C*H), 83.2 (s, Ar′-*C*≡CH), 79.7 (s, Ar-*C*≡CH), 79.7 (d, Ar′-C≡*C*H), 56.0 (q, O*C*H_3_) ppm. HRMS (EI): *m*/*z* = calcd. 411.1371; found 411.1367 (Δ 1.13 ppm). IR (ATR): $\tilde{\nu }$ = 3280, 3246 (C≡C-H), 3008 (aryl-H), 2970 (OCH_3_), 2926 (C-H-val.), 2160 (C≡C), 1606, 1574, 1504, 1408 (arom. C=C, arom. C=N), 1437 (C-H-def.), 1353 (C-N-val.), 812 (1,4-disubst. aryl, 1,3,4-trisubst. aryl) cm^−1^.

### 3.12. 2,4,6-Tris{4-[3,5-bis(ethoxycarbonyl)phenylethynyl]-phenyl}-1,3,5-triazine *(**11a**)*

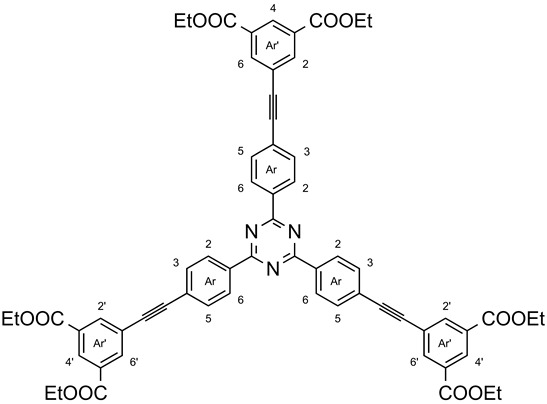



Under nitrogen, 2,4,6-tris(4-ethynylphenyl)-1,3,5-triazine (**10a**, 50.0 mg, 131 µmol) and diethyl 5-iodoisophthalate (**5**, 171 mg, 491 µmol) were dissolved in a mixture of tetrahydrofuran (6 mL) and triethylamine (4 mL). Bis(triphenylphosphine)-palladium(II) dichloride (10 mg, 14 µmol) and copper(I) iodide (3.0 mg, 14 µmol) were added, and the mixture was stirred for 48 h at 50 °C. After evaporation of the solvents in vacuo, chloroform (25 mL) was added to the residue. The organic layer was washed with deionized water (3 × 25 mL) and brine (25 mL). After drying with magnesium sulfate and filtration, the solvent was removed in vacuo. The crude product was recrystallized from a boiling mixture of toluene and *n*-heptane, yielding 108 mg (104 umol, 79%) of a yellowish solid, m. p. >300 °C. ^1^H NMR (500 MHz, CDCl_3_): δ = 8.75 (d, 6 H, *J* = 8.4 Hz, Ar-*H*-2,6), 8.64 (t, 3 H, *J* = 1.6 Hz, Ar′-*H*-4), 8.39 (d, 6 H, *J* = 1.6 Hz, Ar′-*H*-2,6), 7.74 (d, 6 H, *J* = 8.4 Hz, Ar-*H*-3,5), 4.44 (q, 12 H, *J* = 7.1 Hz, CO_2_C*H*_2_CH_3_), 1.45 (t, 18 H, *J* = 7.1 Hz, CO_2_CH_2_C*H*_3_) ppm. ^13^C NMR (125 MHz, CDCl_3_): δ = 171.0 (s, tri-*C*-2,4,6), 165.1 (s, *C*O_2_Et), 136.5 (d, Ar′-*C*-2,6), 136.0 (s, Ar-*C*-1), 132.0 (d, Ar-*C*-3,5), 131.4 (s, Ar′-*C*-3,5), 130.3 (d, Ar′-*C*-4), 129.0 (d, Ar-*C*-2,6), 126.9 (s, Ar-*C*-4), 123.9 (s, Ar′-*C*-1), 90.9 (s, Ar-*C*≡C-Ar′), 90.3 (s, Ar-C≡*C*-Ar′), 61.6 (t, CO_2_*C*H_2_CH_3_), 14.3 (q, CO_2_CH_2_*C*H_3_) ppm. MS (MALDI, Cl-CCA): *m*/*z* = 1042 [M + H]^+^. IR (ATR): $\tilde{\nu }$ = 3005 (aryl-H), 2985 (C-H-val.), 1690 (C=O), 1607, 1509 (arom. C=C, arom. C=N), 1429 (CH-def.), 1356 (C-N-val.), 825 (1,4-disubst. aryl, 1,3,5-trisubst. aryl) cm^−1^. Elemental analysis (C_63_H_51_N_3_O_12_) (1042.04): calcd. C 72.61 H 4.93 N 4.03; (C_63_H_51_N_3_O_12_·0.3 CDCl_3_) (1077.85): calcd. C 70.53 H 4.80 N 3.90; found C 70.20 H 4.68 N 4.28.

### 3.13. 2-{4-[3,5-Bis(ethoxycarbonyl)phenylethynyl]-3-methoxy-phenyl}-4,6-bis{4-[3,5-bis(ethoxycarbonyl)phenylethynyl]-phenyl}-1,3,5-triazine *(**11c**)*

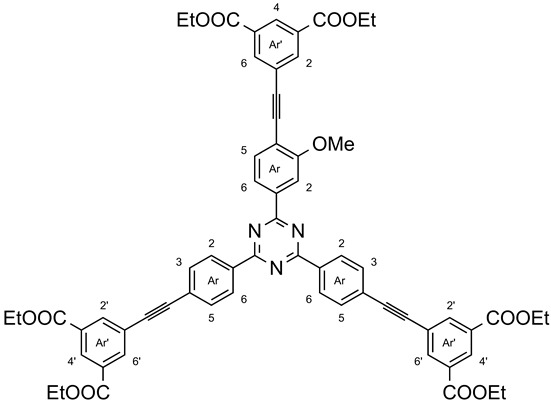



Under nitrogen, 2,4-bis(4-ethynylphenyl)-6-(3-methoxy-4-ethynylphenyl)-1,3,5-triazine (**10c**, 50.0 mg, 122 µmol) and diethyl 5-iodoisophthalate (**5**, 159 mg, 456 µmol) were dissolved in a mixture of tetrahydrofuran (6 mL) and triethylamine (4 mL). After the addition of bis(triphenylphosphine)-palladium(II) dichloride (10 mg, 14 µmol) and copper(I) iodide (3.0 mg, 14 µmol), the mixture was stirred at 50 °C for 48 h. After evaporation of the volatiles, chloroform (25 mL) was added, and the mixture was extracted with deionized water (3 × 25 mL) and brine (25 mL). The organic layer was dried with magnesium sulfate, filtered, and the solvent was evaporated in vacuo. The crude product was recrystallized from a boiling mixture of toluene *n*-heptane, yielding 106 mg (98.8 μmol, 81%) of a yellowish solid, m. p. >300 °C. ^1^H NMR (500 MHz, CDCl_3_): δ = 8.79 (d, 4 H, *J* = 8.2 Hz, Ar′-*H*-2,6), 8.66 (s, 2 H, Ar′-*H*-4′), 8.65 (s, 1 H, Ar-*H*-4′), 8.44 (d, 2 H, *J* = 1.2 Hz, Ar-*H*-2′,6′), 8.43–8.41 (m, 5 H, Ar′-*H*-2′,6′, Ar-*H*-6), 8.35 (s, 1 H, Ar-*H*-2), 7.77 (d, 4 H, *J* = 8.2 Hz, Ar′-*H*-3,5), 7.72 (d, 1 H, *J* = 7.9 Hz, Ar-*H*-5), 4.48–4.42 (m, 12 H, OC*H*_2_CH_3_), 4.18 (s, 3 H, OC*H*_3_), 1.48–1-39 (m, 18 H, OCH_2_C*H*_3_) ppm. ^13^C NMR (125 MHz, CDCl_3_): δ = 171.1 (s, tri-*C*-2), 171.0 (s, tri-*C*-4,6), 165.2 (s, Ar′-*C*OEt), 165.1 (s, Ar-*C*OEt), 161.0 (s, Ar-*C*-3), 136.5 (d, Ar-*C*-2′,6′), 136.5 (s, Ar-*C*-1), 136.0 (s, Ar′-*C*-1), 135.3 (d, Ar′-*C*-2′,6′), 133.9 (d, Ar-*C*-5), 132.1 (d, Ar′-*C*-3,5), 131.4 (s, Ar′-*C*-3′,5′), 131.3 (s, Ar-*C*-3′,5′), 130.3 (d, Ar′-*C*-4′), 130.0 (d, Ar-*C*-4), 129.0 (d, Ar′-*C*-2,6), 127.0 (s, Ar′-*C*-4), 121.4 (d, Ar-*C*-6), 116.2 (s, Ar-*C*-4), 110.9 (d, Ar-*C*-2), 94.0 (s, Ar-C≡*C*), 90.7 (s, Ar′-*C*≡C), 90.1 (s, Ar′-C≡*C*), 87.8 (Ar-*C*≡C), 61.7 (t, Ar′-CO-*C*H_2_CH_3_), 61.6 (t, Ar-CO-*C*H_2_CH_3_), 56.1 (q, O*C*H_3_), 14.4 (q, OCH_2_*C*H_3_) ppm. IR (ATR): $\tilde{\nu }$ = 3075 (aryl-H), 2985, 2941 (C-H-val.), 1721 (C=O), 1599, 1569 (arom. C=C, arom. C=N), 1444 (CH-def.), 1366 (C-N-val.), 810 (1,4-disubst. aryl, 1,3,5-trisubst. aryl) cm^−1^. MS (MALDI, Cl-CCA): *m*/*z* = 1073 [M + H]^+^.

## 4. Conclusions

Extended, mono-substituted triazine-based hexacarboxylates have been synthesized starting from mono-nitro and mono-methoxy substituted 2,4,6-tris(4-bromophenyl)-1,3,5-triazines **3b** and **c**. Five new extended hexaesters were obtained by either Suzuki-Miyaura coupling (**6**) or two subsequent Sonogashira-Hagihara couplings (**11**) in good yields and large quantities (up to >1 g). Hydrolyses of the hexaesters **6** to give hexacarboxylic acids **7** were successful. Five of the new linkers carry an additional functional group in one of the arms (NO_2_ or OMe). The extended linkers **6**, **7**, or **11** must now be employed in MOF syntheses, in the hope that the stiff structure of the linkers allows the formation of isoreticular structures, as already observed for tridentate triazine–based linkers [14]. The influence of the additional substituent, nitro or methoxy, on MOF formation and on MOF properties has to be studied. Further modification of the substituents should also be possible. In the tridentate analogues, for instance **2**, reduction of the nitro group to give an amino group and cleavage of the methoxy group to give a hydroxy function were successful [13]. 

## Supplementary Materials

^1^H and ^13^C-NMR spectra of compounds **6**, **7**, **9**, **10**, and **11** are available online.
