# Supplementary material for: Substituted 1,3,5-Triazine Hexacarboxylates as Potential Linkers for MOFs
Source: Molecules. 2019 Sep 25;24(19):3480. doi: 10.3390/molecules24193480 (PMC6804050; doi:10.3390/molecules24193480)
Supplement: Supplementary file 1 [file molecules-24-03480-s001.pdf]

Substituted triazine hexacarboxylates as linkers for MOFs

Arne Klinkebiel, Ole Beyer, Ulrich Lüning*

* Otto-Diels-Institut für Organische Chemie, Christian-Albrechts-Universität zu Kiel, Olshausenstr. 40, D-24098 Kiel, Germany

Supporting information: NMR spectra for

**6a**	p. 2
**6b**	p. 4
**6c**	p. 6
**7a**	p. 8
**7b**	p. 10
**7c**	p. 12
**9a**	p. 14
**9b**	p. 16
**9c**	p. 18
**10a**	p. 20
**10c**	p. 22
**11a**	p. 24
**11c**	p. 26

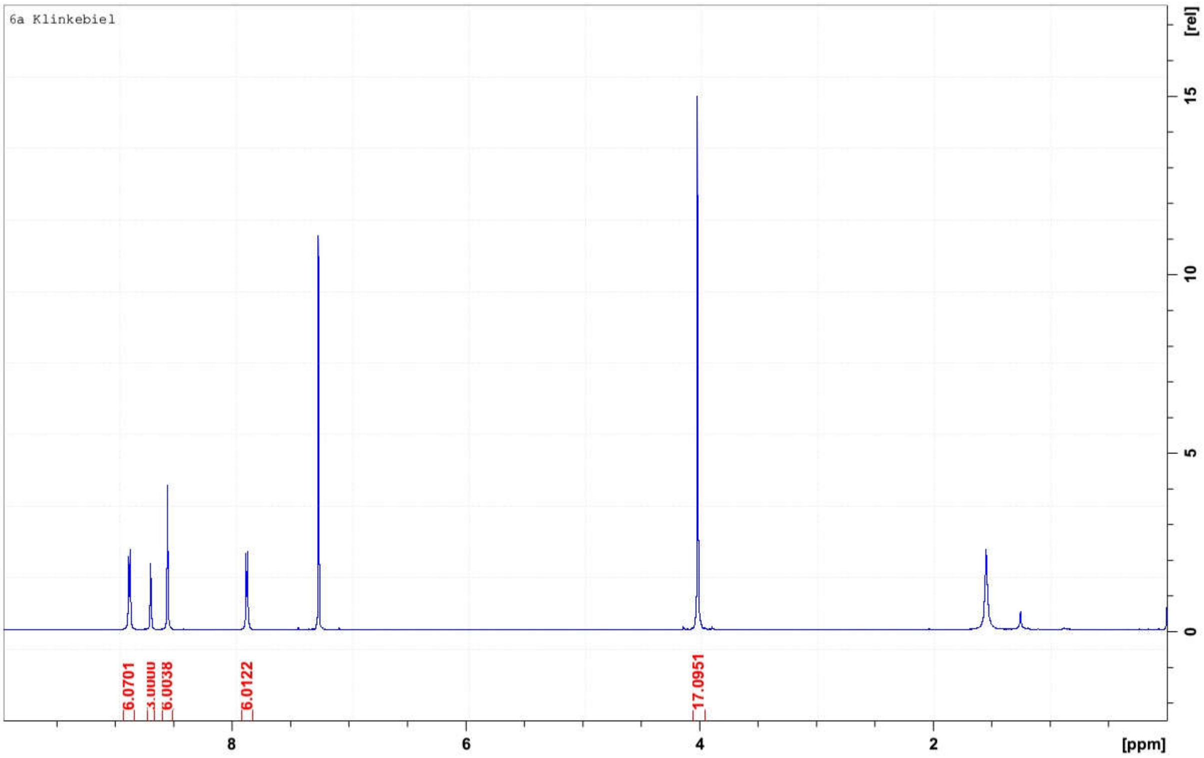

Figure S1. ^{1}H NMR spectrum of **6a**.

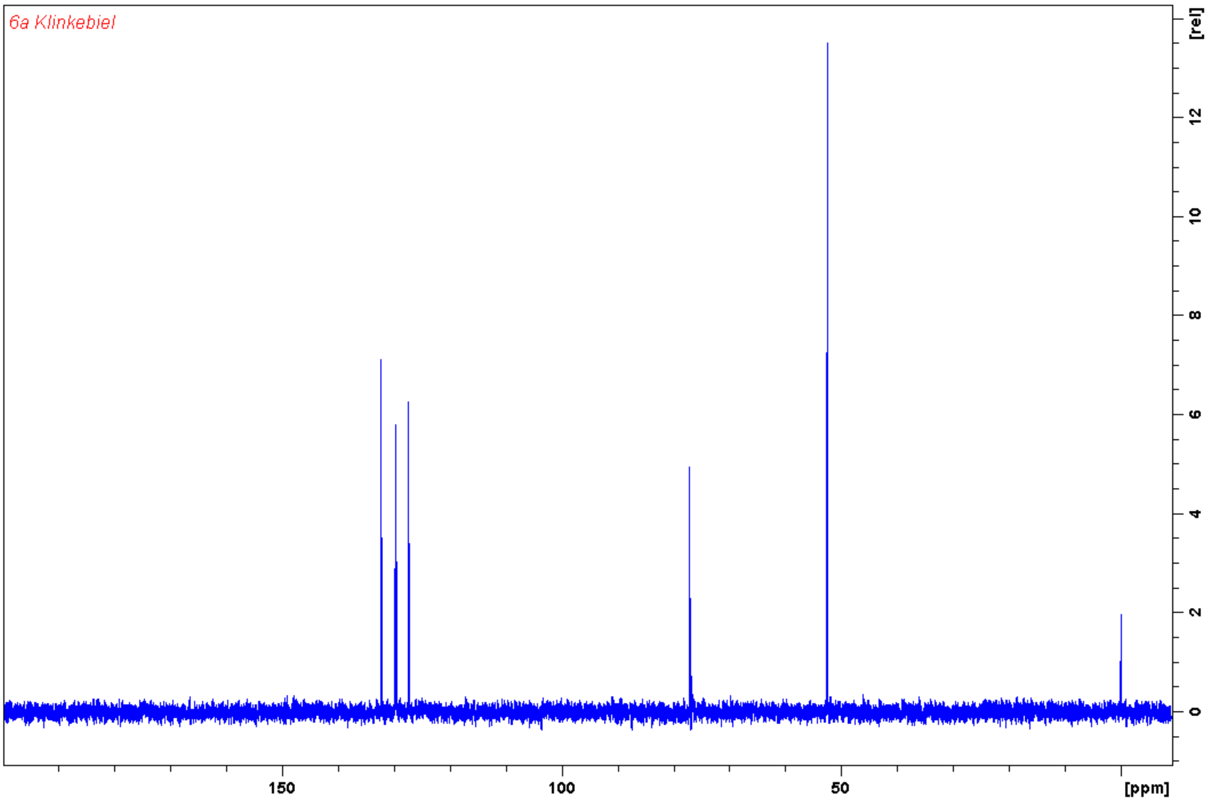

Figure S2. ^{13}C NMR spectrum of **6a**.

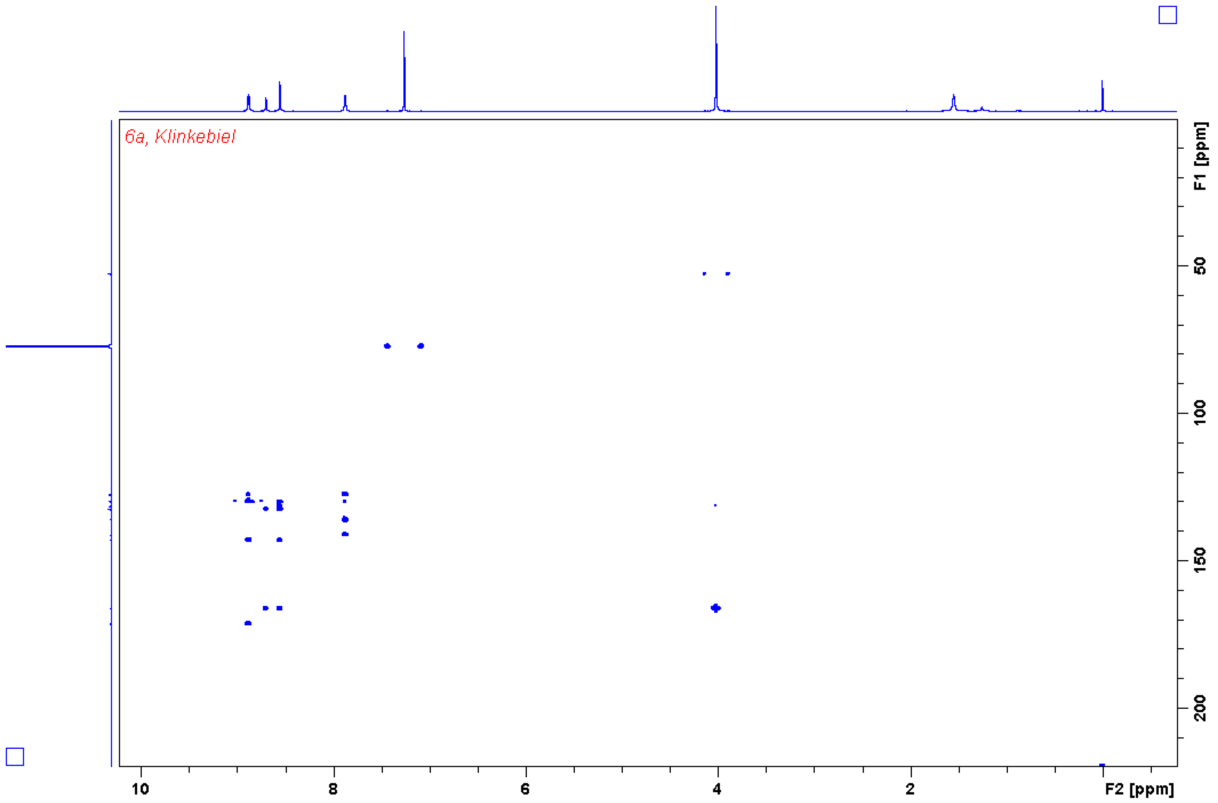

Figure S3. HMBC NMR spectrum of **6a**.

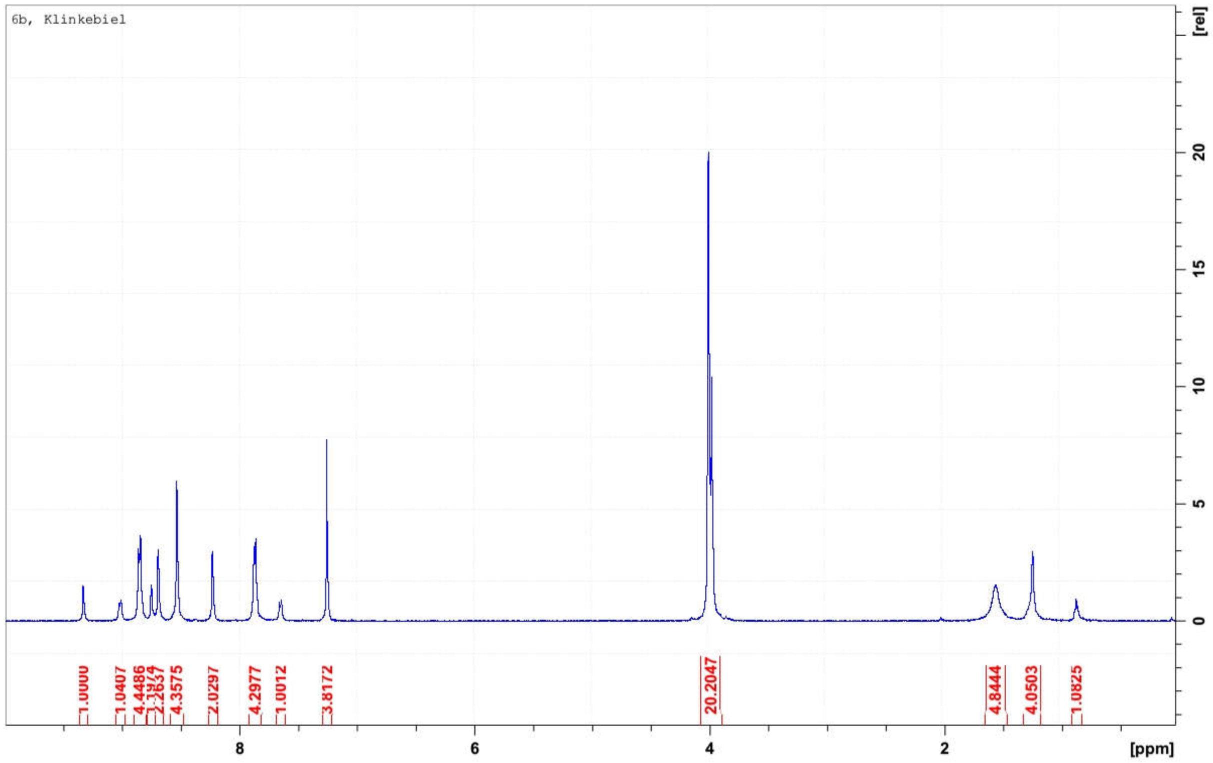

Figure S4. ^{1}H NMR spectrum of **6b**.

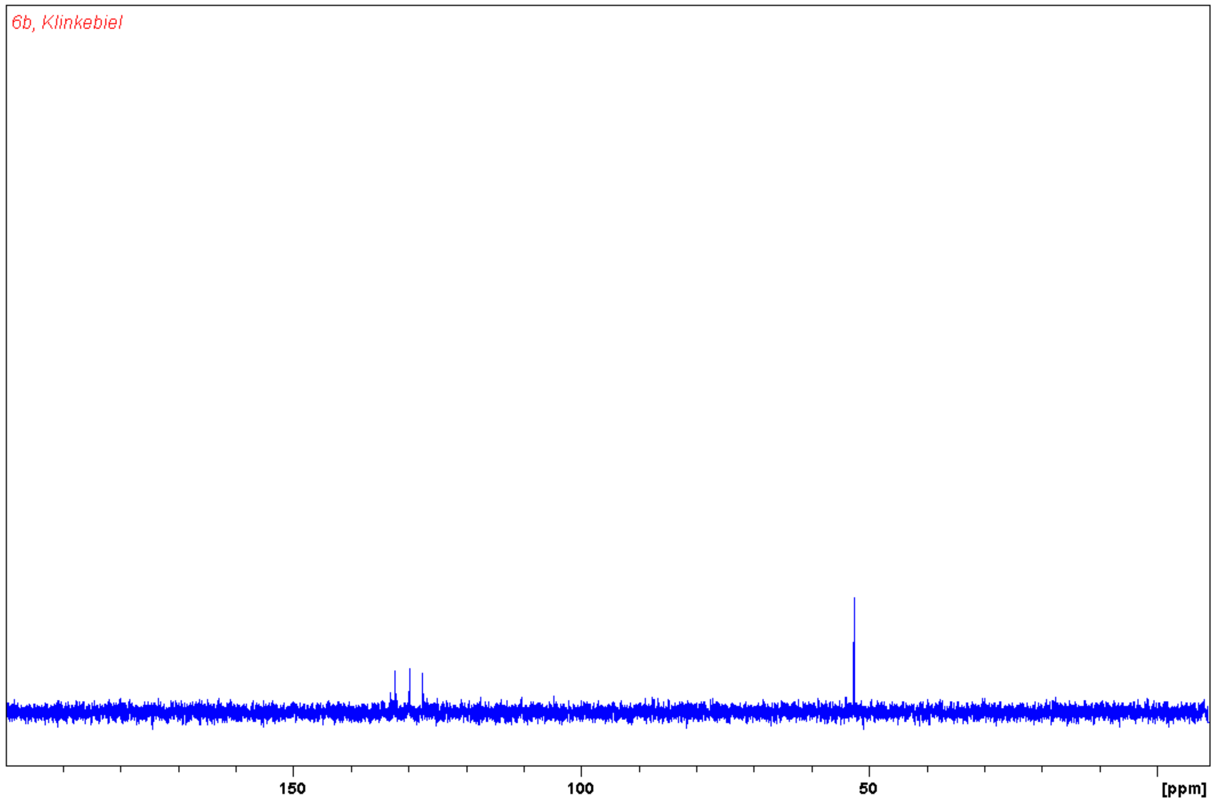

Figure S5. ^{13}C NMR spectrum of **6b**.

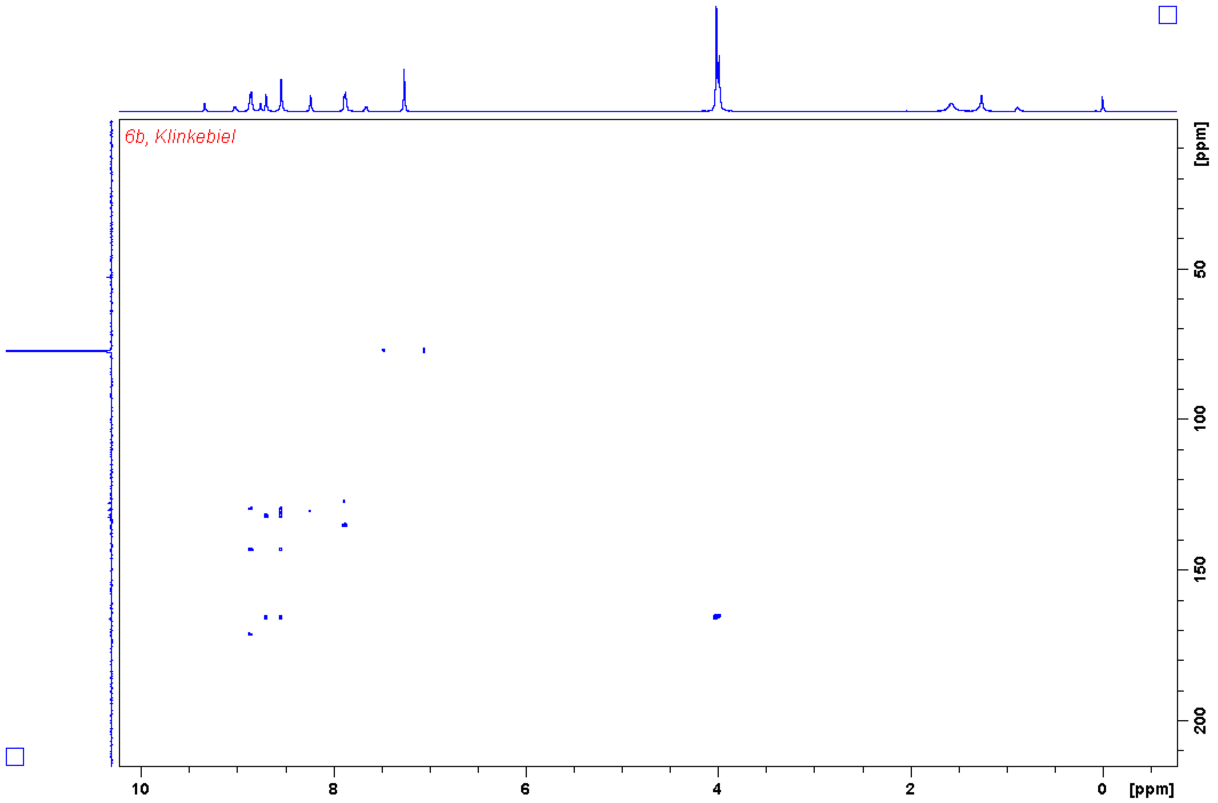

Figure S6. HMBC NMR spectrum of **6b**.

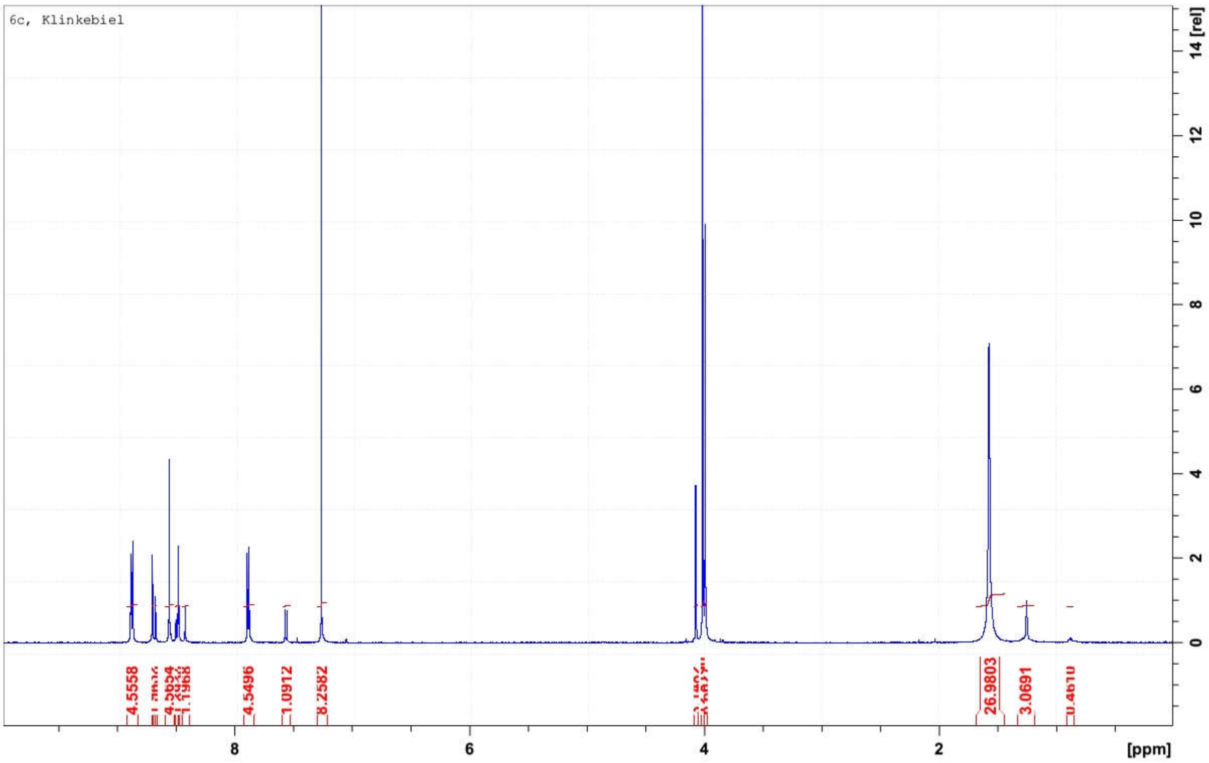

Figure S7. ^{1}H NMR spectrum of **6c**.

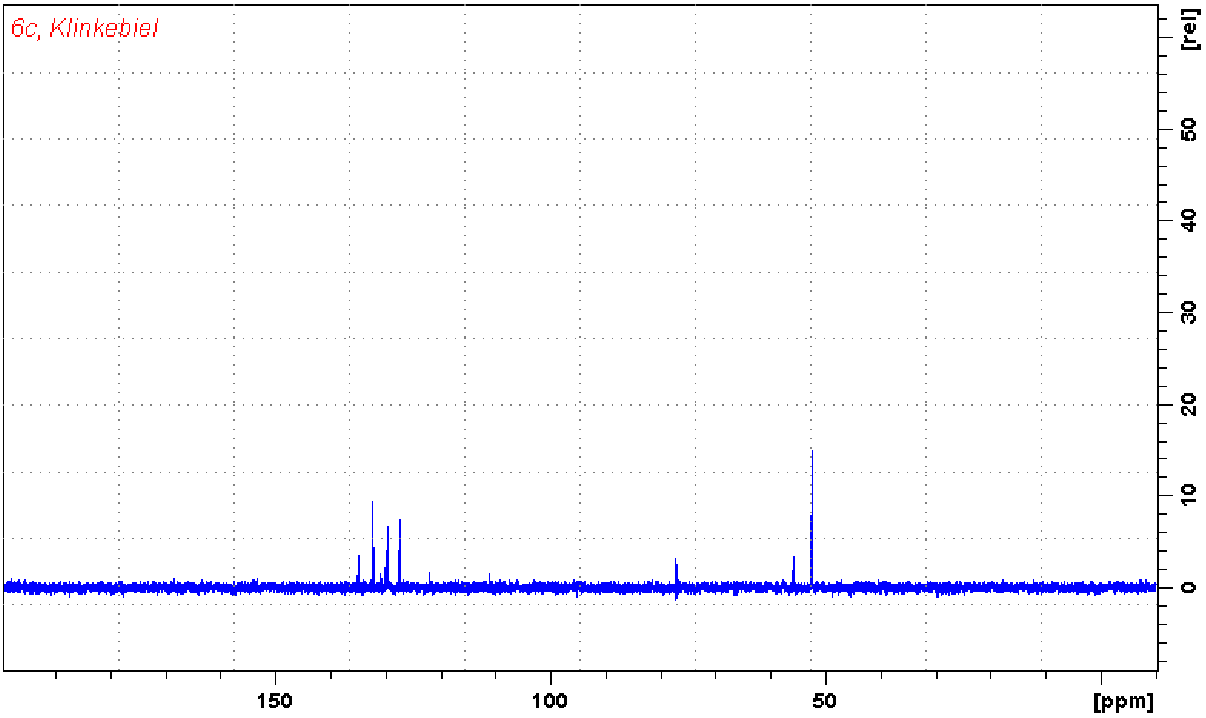

Figure S8. ^{13}C NMR spectrum of **6c**.

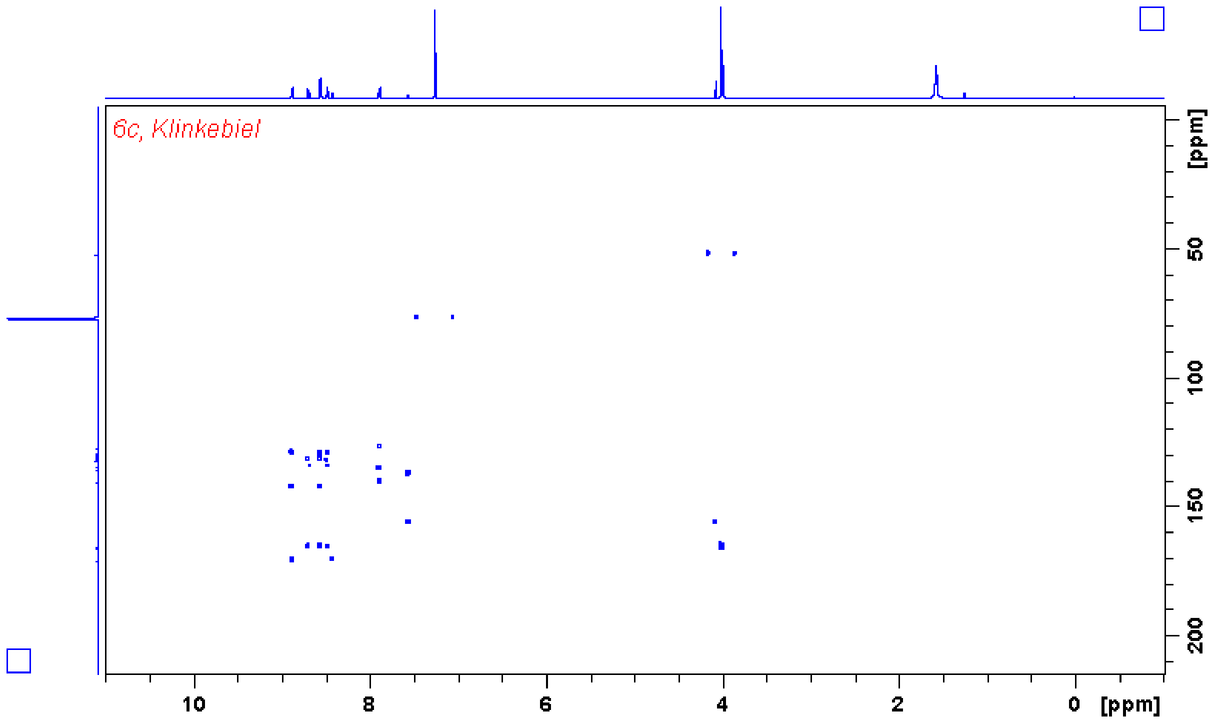

Figure S9. HMBC NMR spectrum of **6c**.

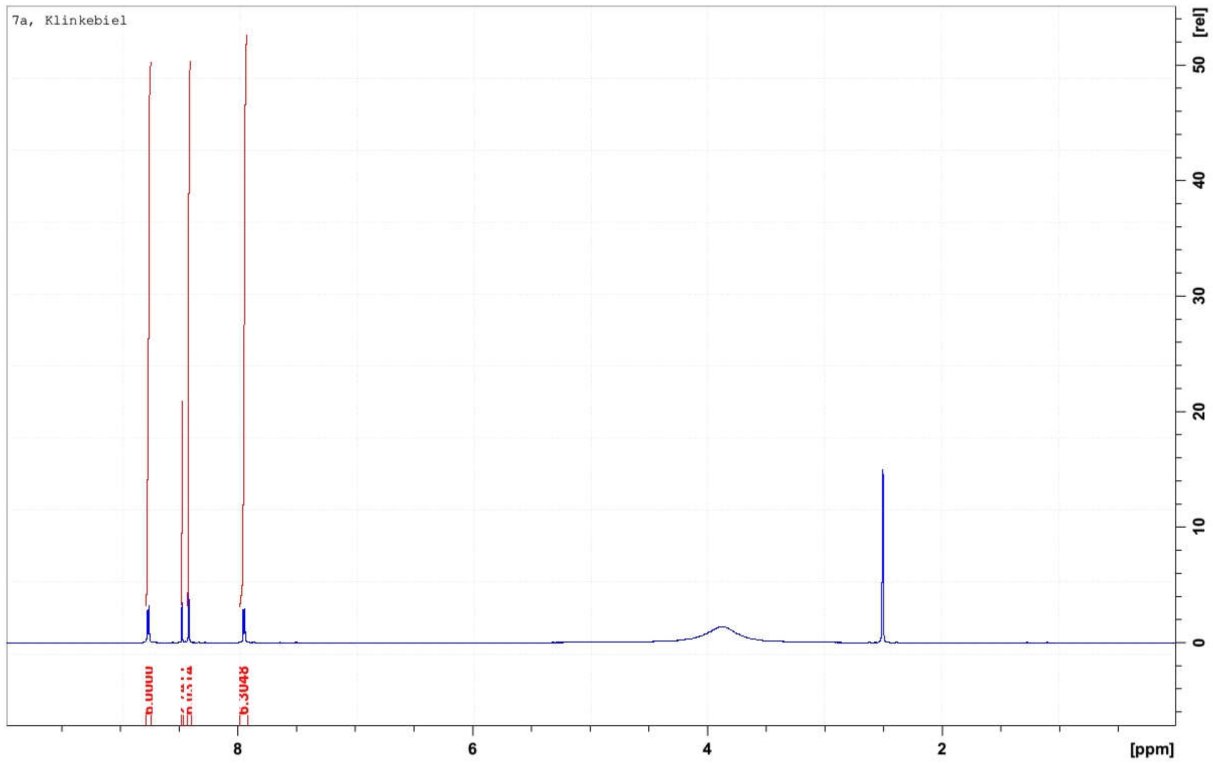

Figure S10. ^{1}H NMR spectrum of **7a**.

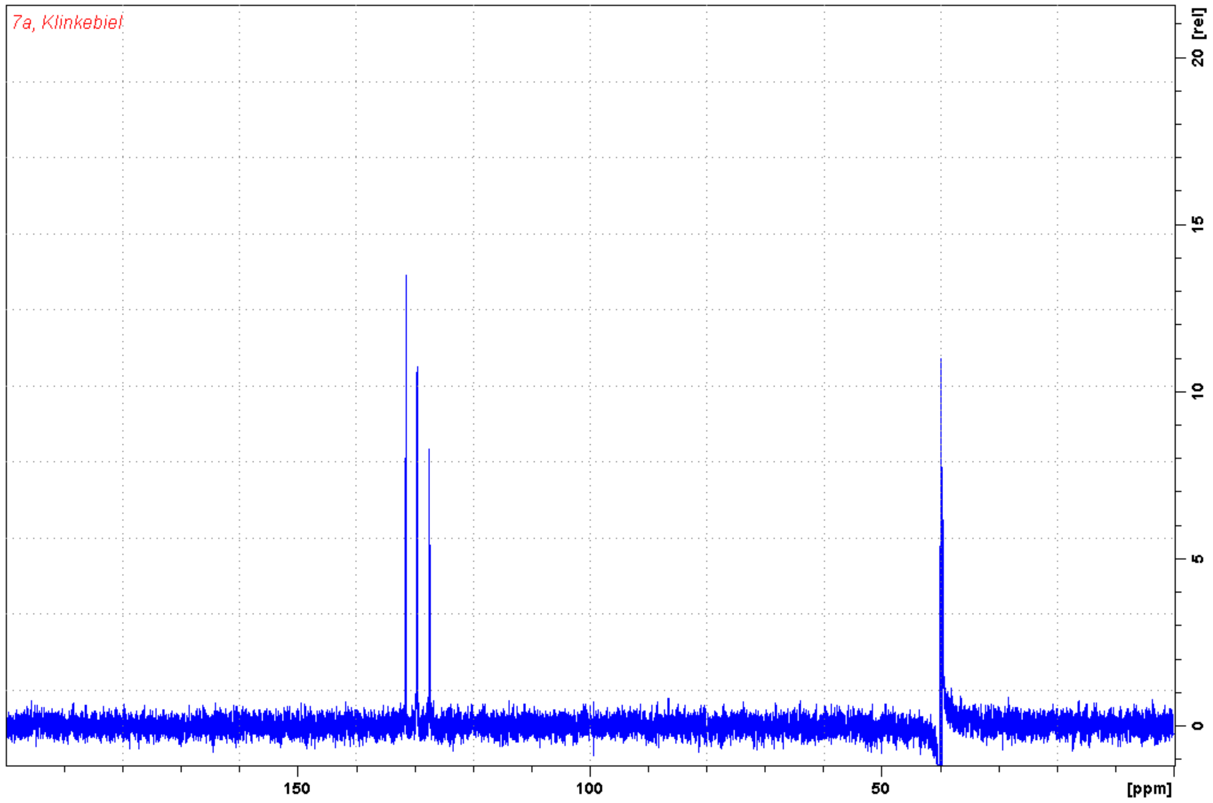

Figure S11. ^{13}C NMR spectrum of **7a**.

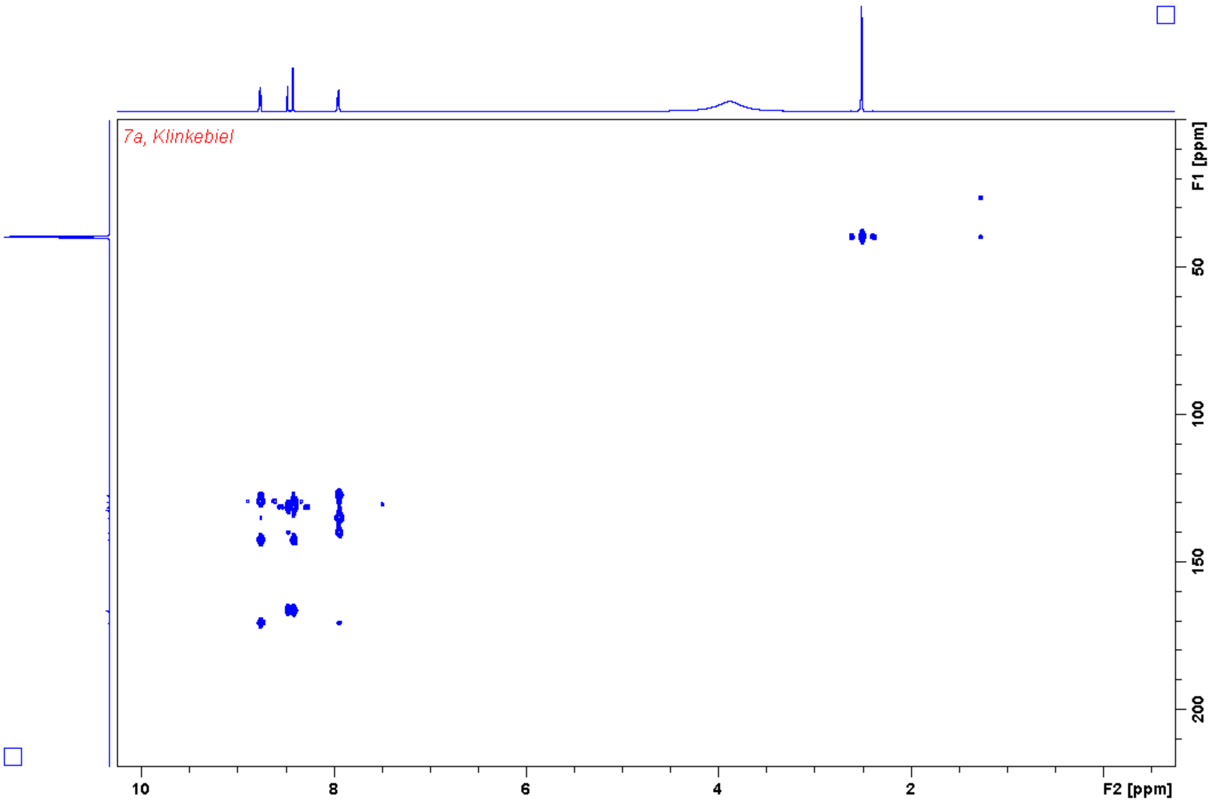

Figure S12. HMBC NMR spectrum of **7a**.

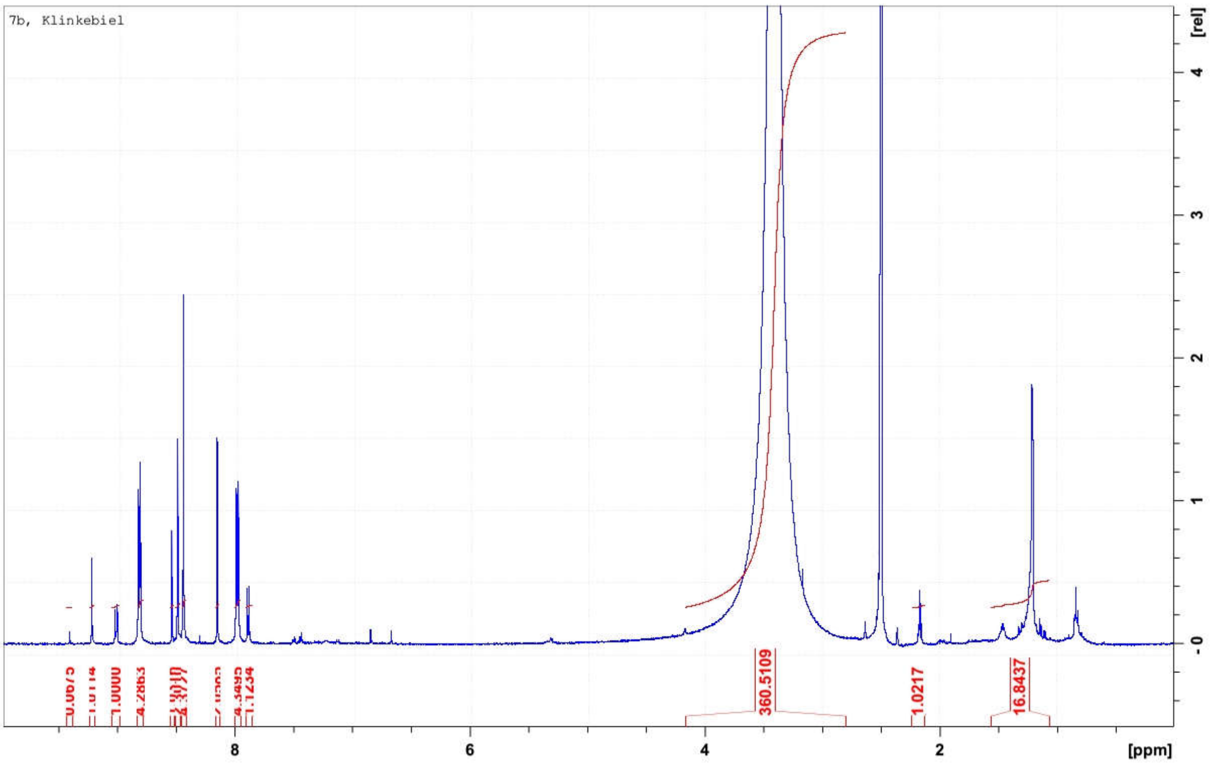

Figure S13. ¹H NMR spectrum of **7b**.

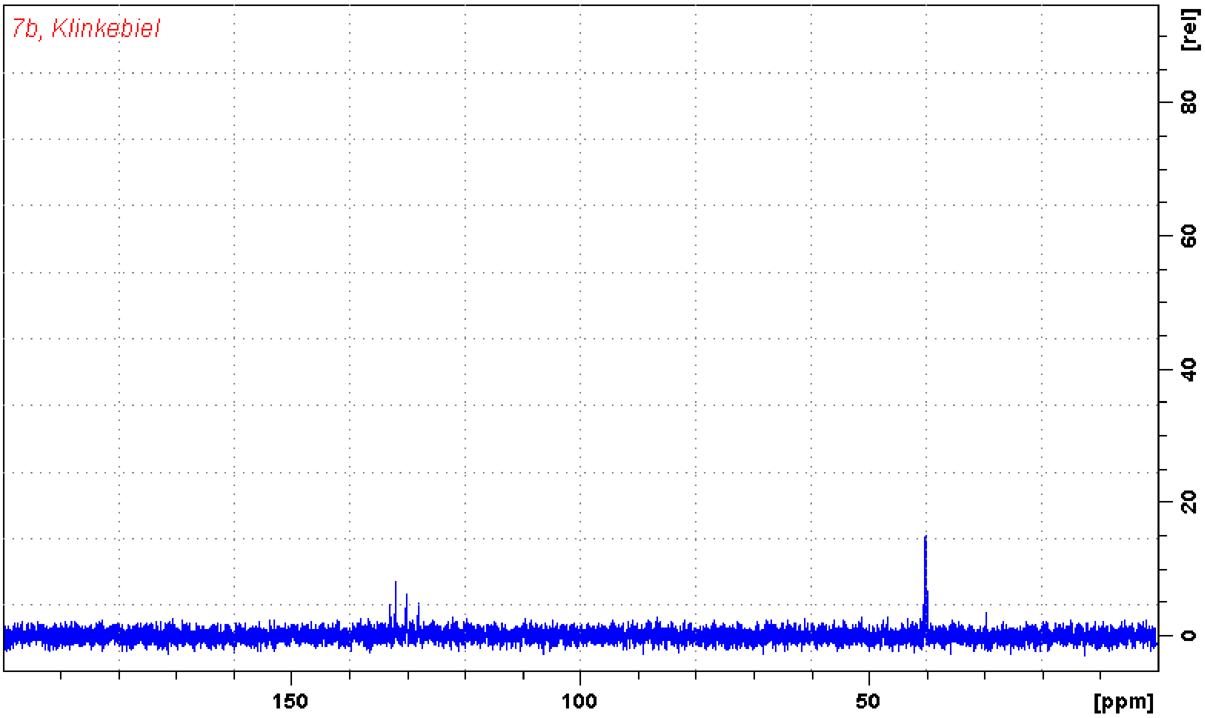

Figure S14. ¹³C NMR spectrum of **7b**.

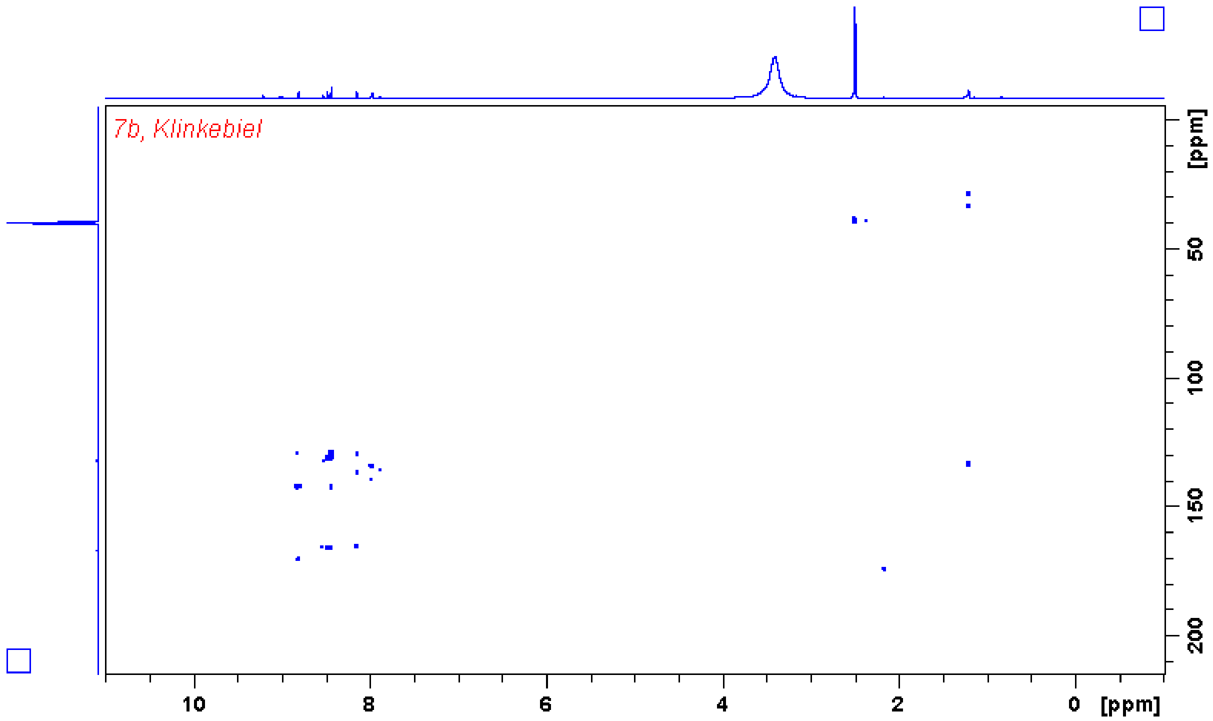

Figure S15. HMBC NMR spectrum of **7b**.

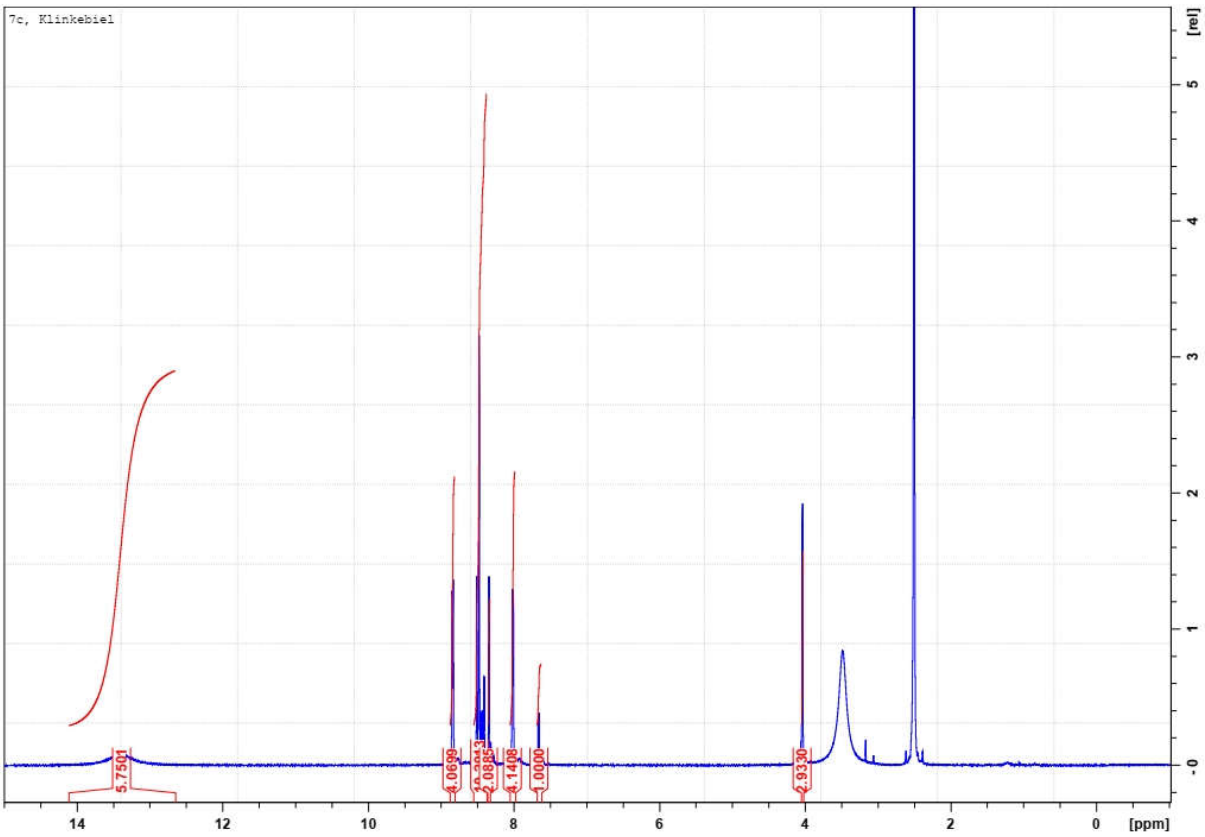

Figure S16. ^{1}H NMR spectrum of **7c**.

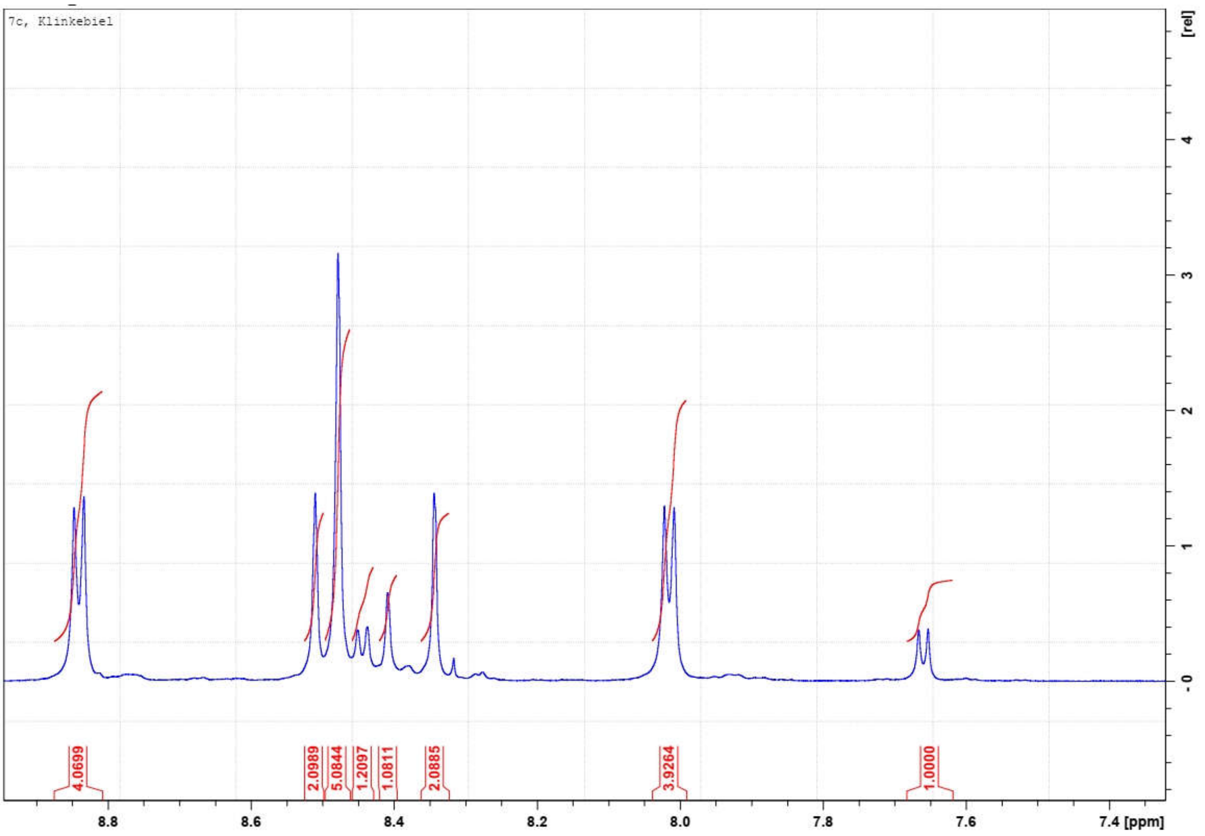

Figure S16a. Aromatic part of ^{1}H NMR spectrum of **7c**.

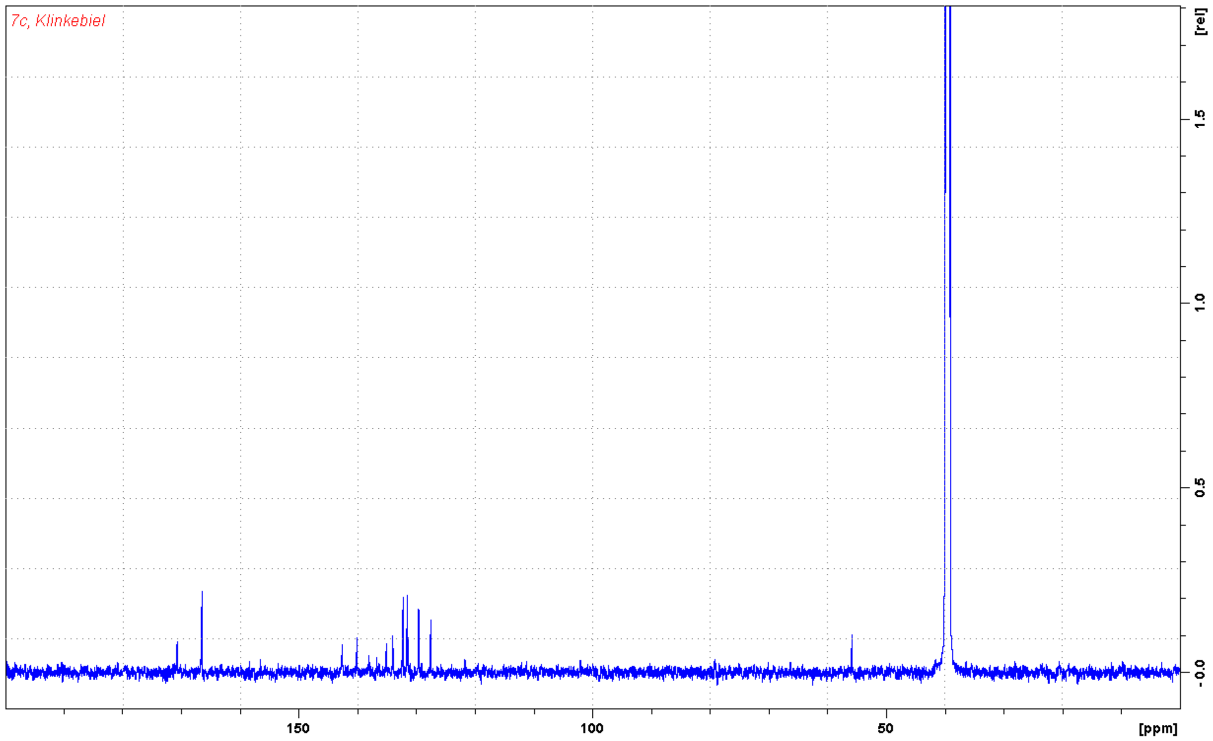

Figure S17. ^{13}C NMR spectrum of **7c**.

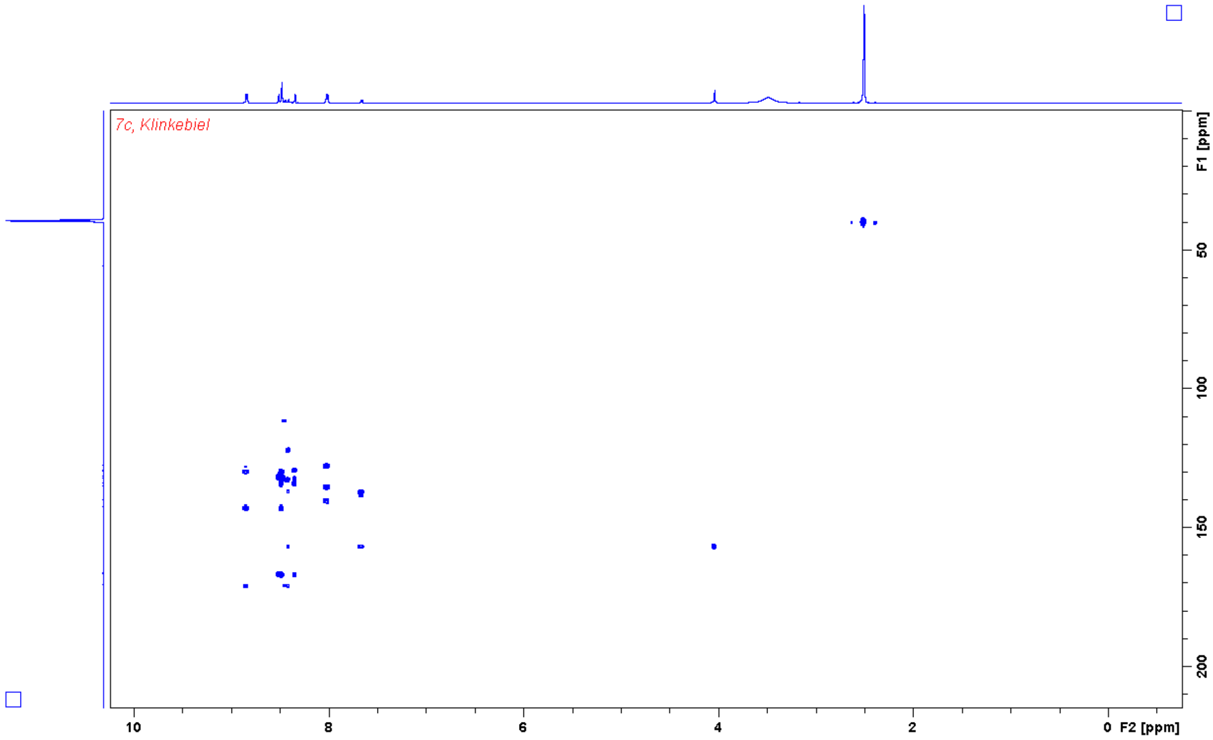

Figure S18. HMBC NMR spectrum of **7c**.

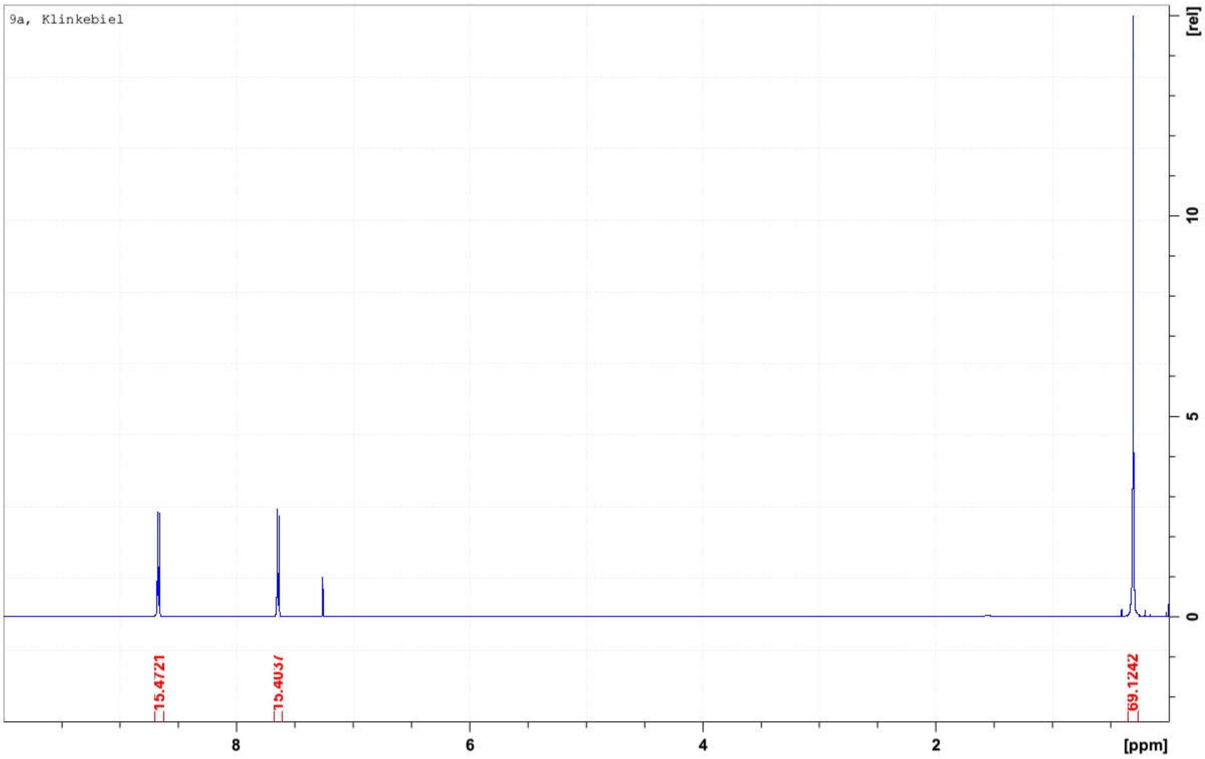

Figure S19. ^{1}H NMR spectrum of **9a**.

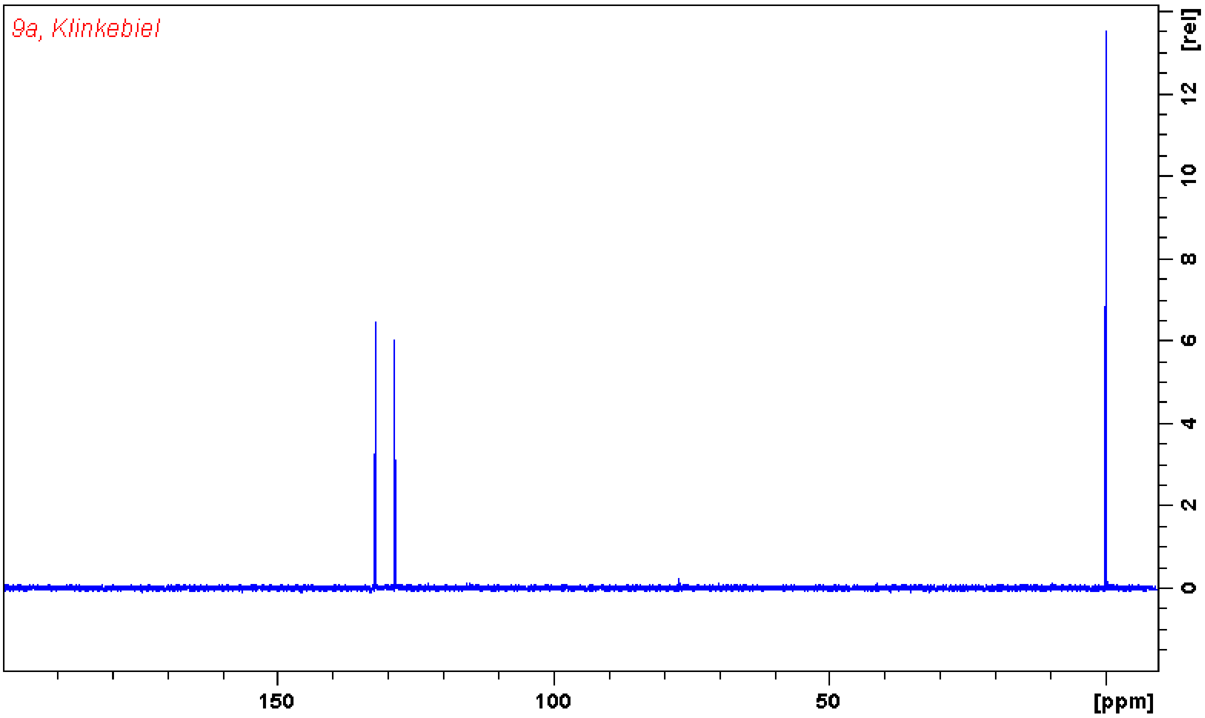

Figure S20. ^{13}C NMR spectrum of **9a**.

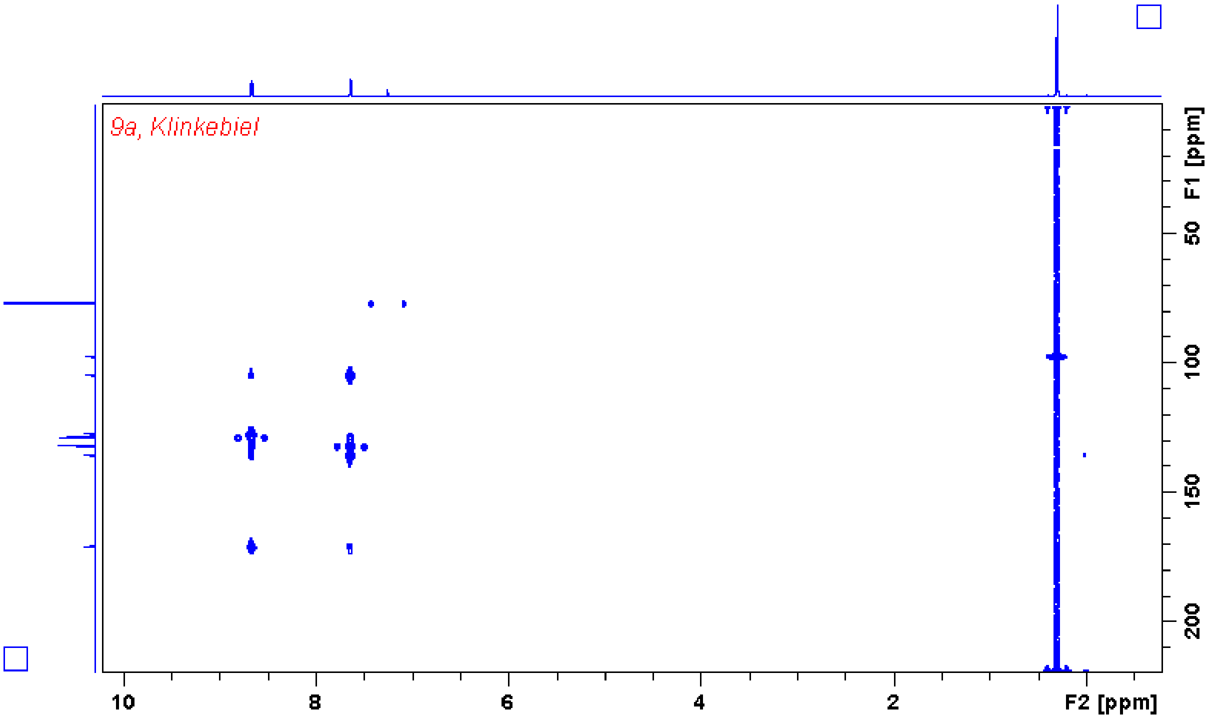

Figure S21. HMBC NMR spectrum of **9a**.

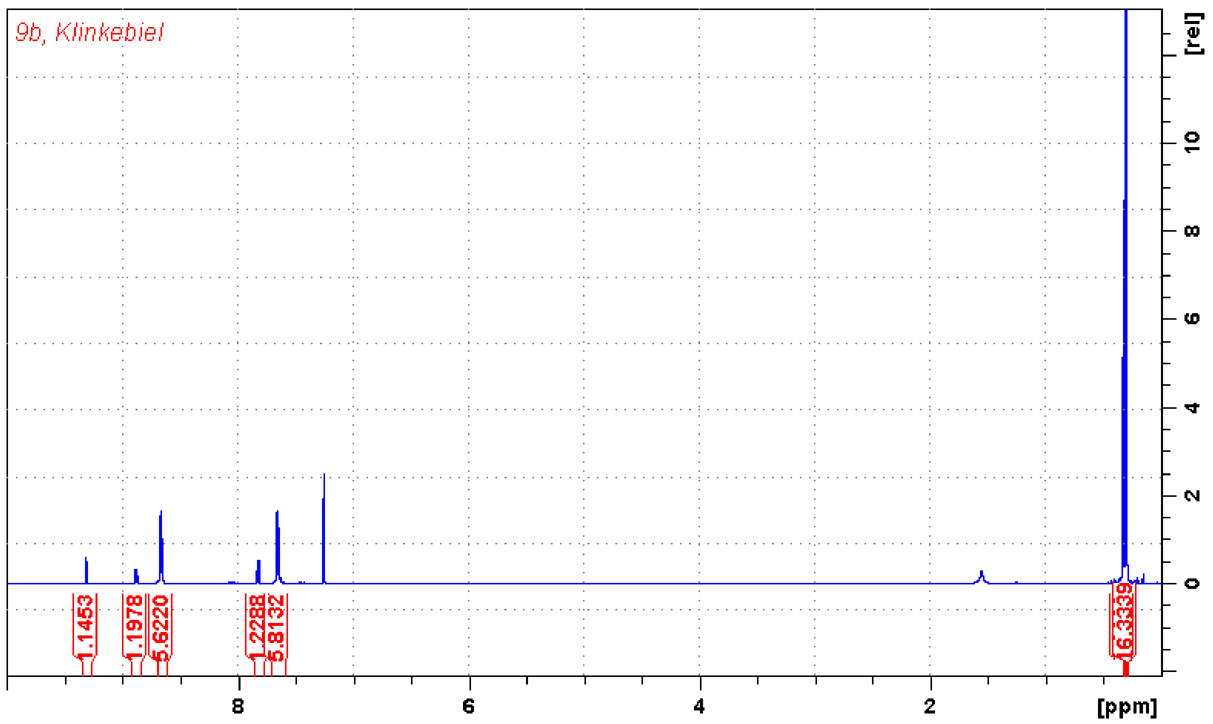

Figure S22. ^{1}H NMR spectrum of **9b**.

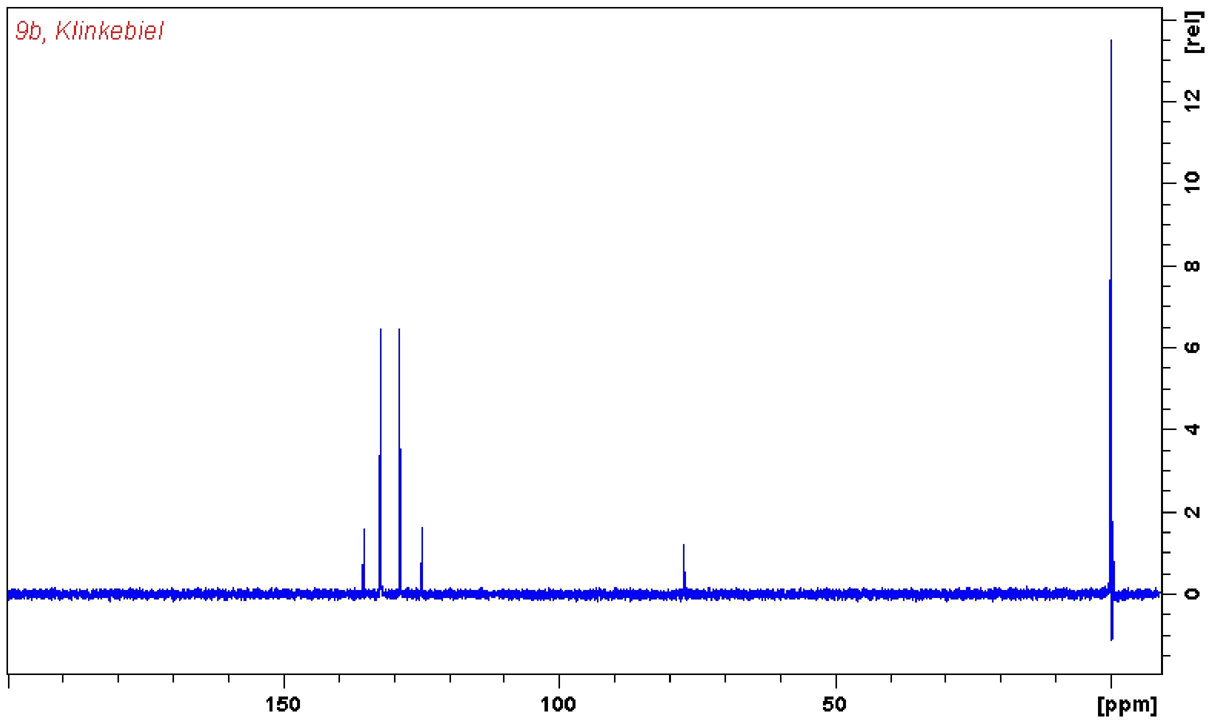

Figure S23. ^{13}C NMR spectrum of **9b**.

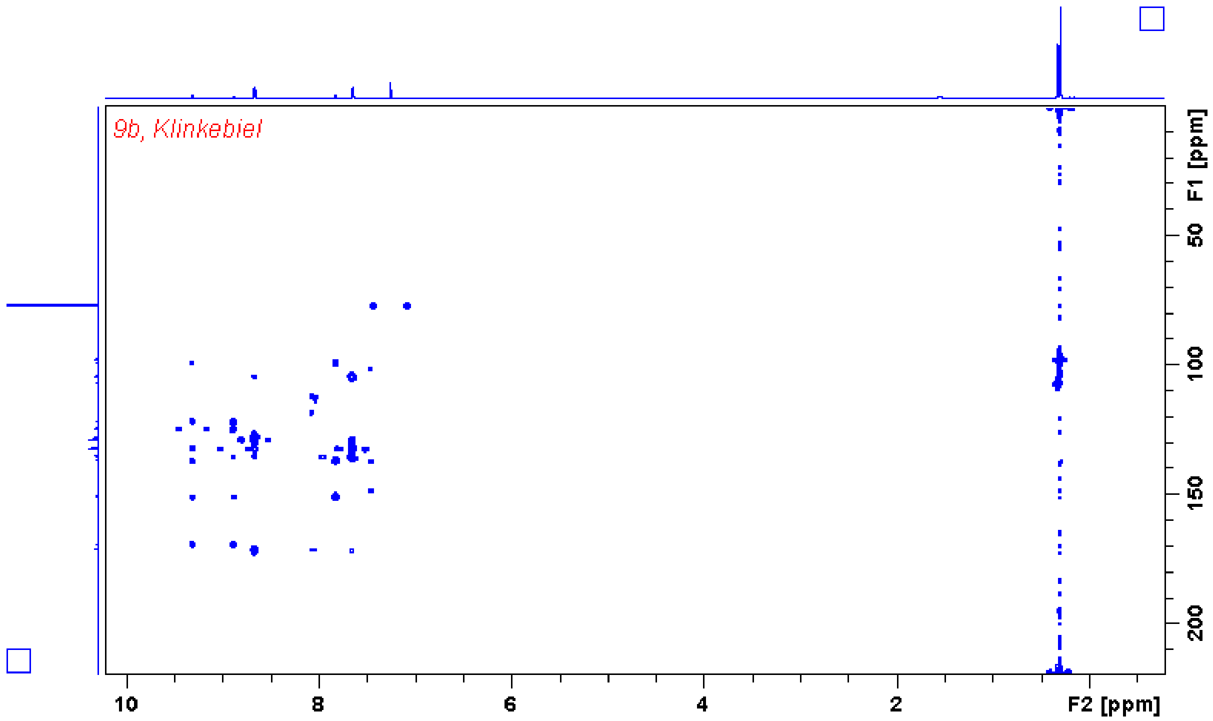

Figure S24. HMBC NMR spectrum of **9b**.

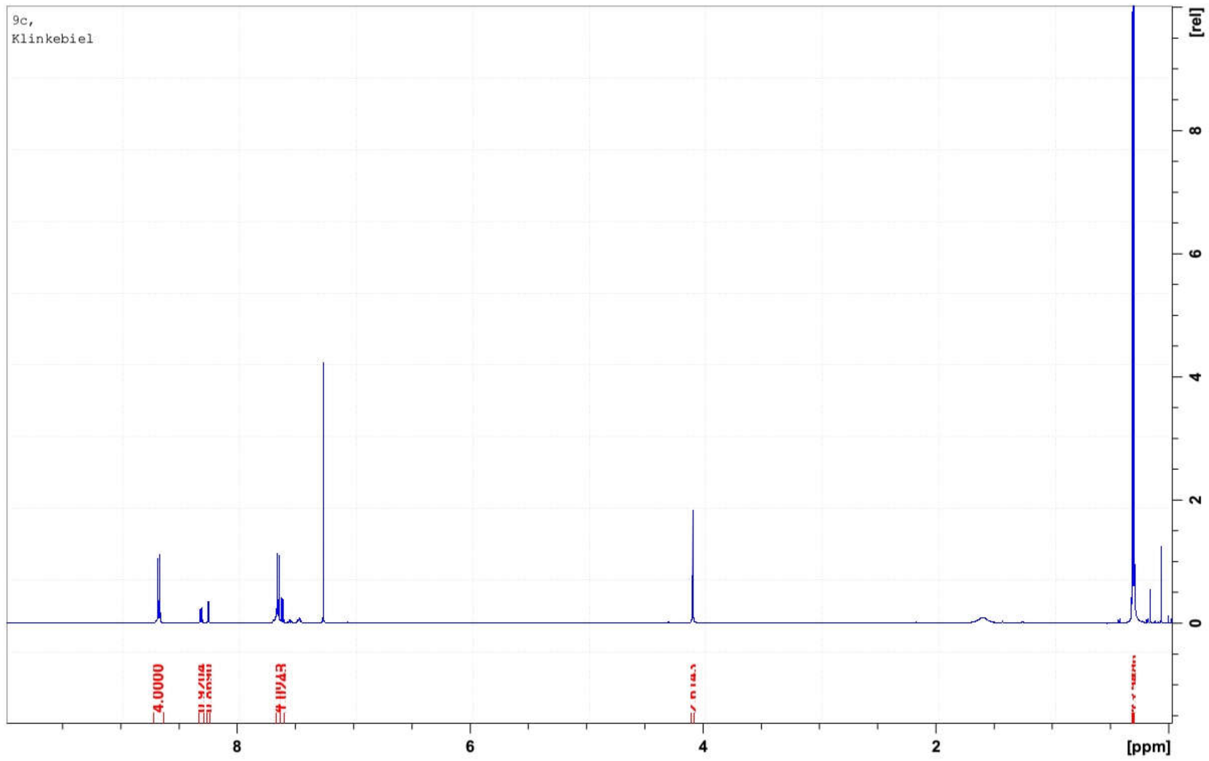

Figure S25. ^{1}H NMR spectrum of **9c**.

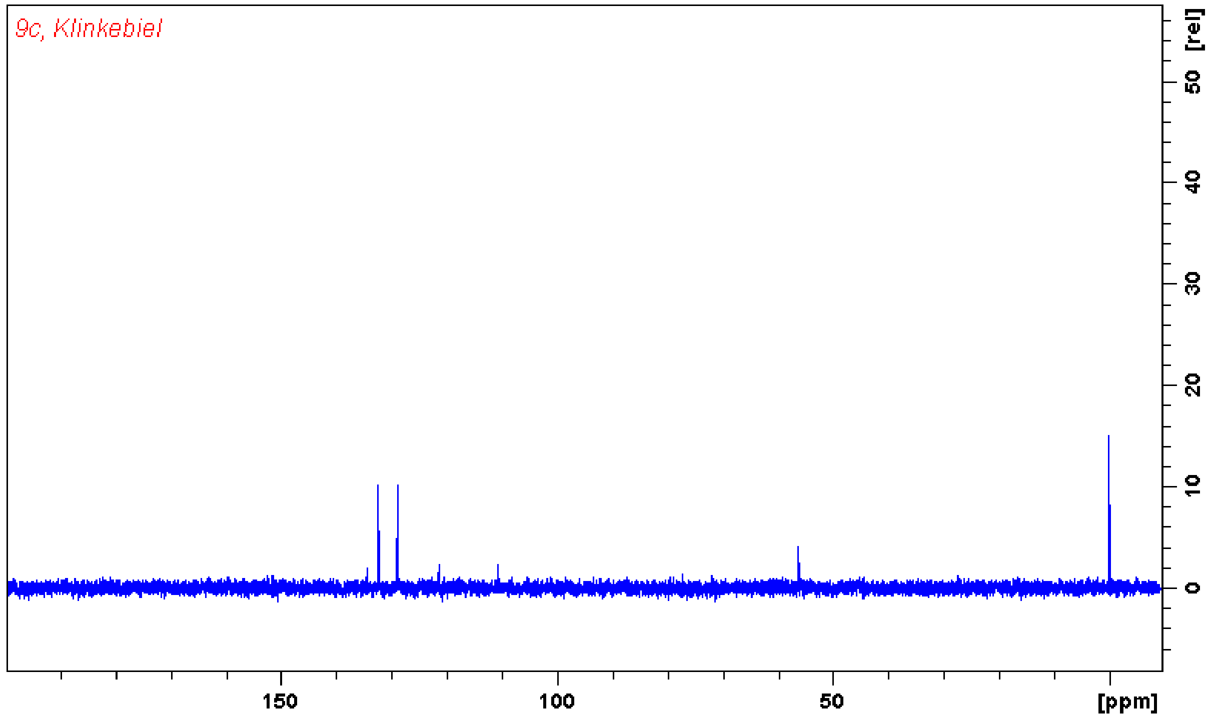

Figure S26. ^{13}C NMR spectrum of **9c**.

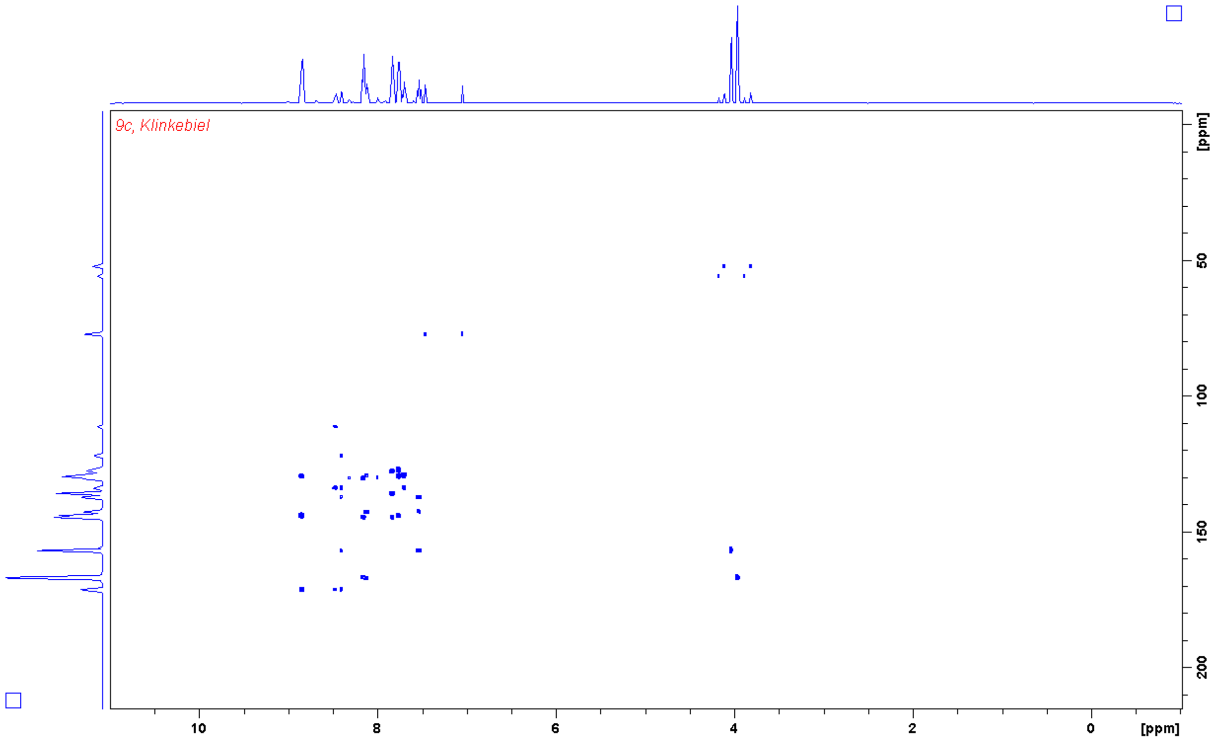

Figure S27. HMBC NMR spectrum of **9c**.

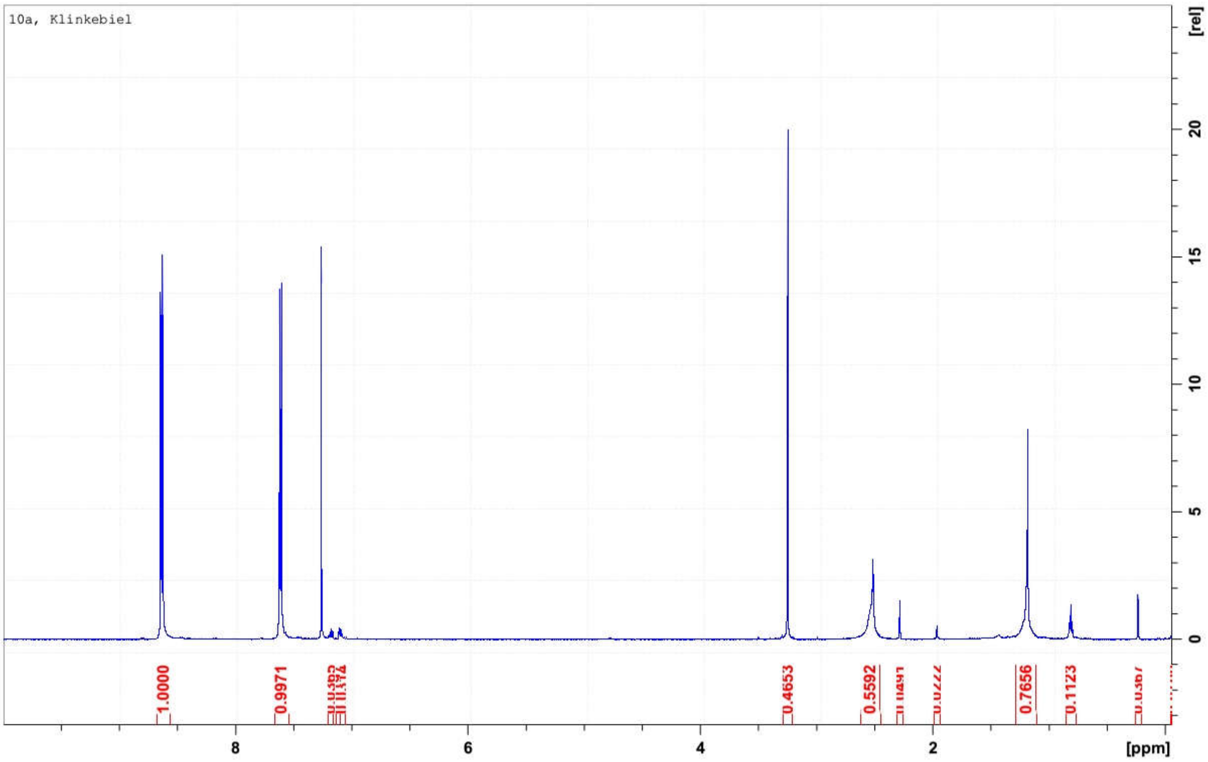

Figure S28. ^{1}H NMR spectrum of **10a**.

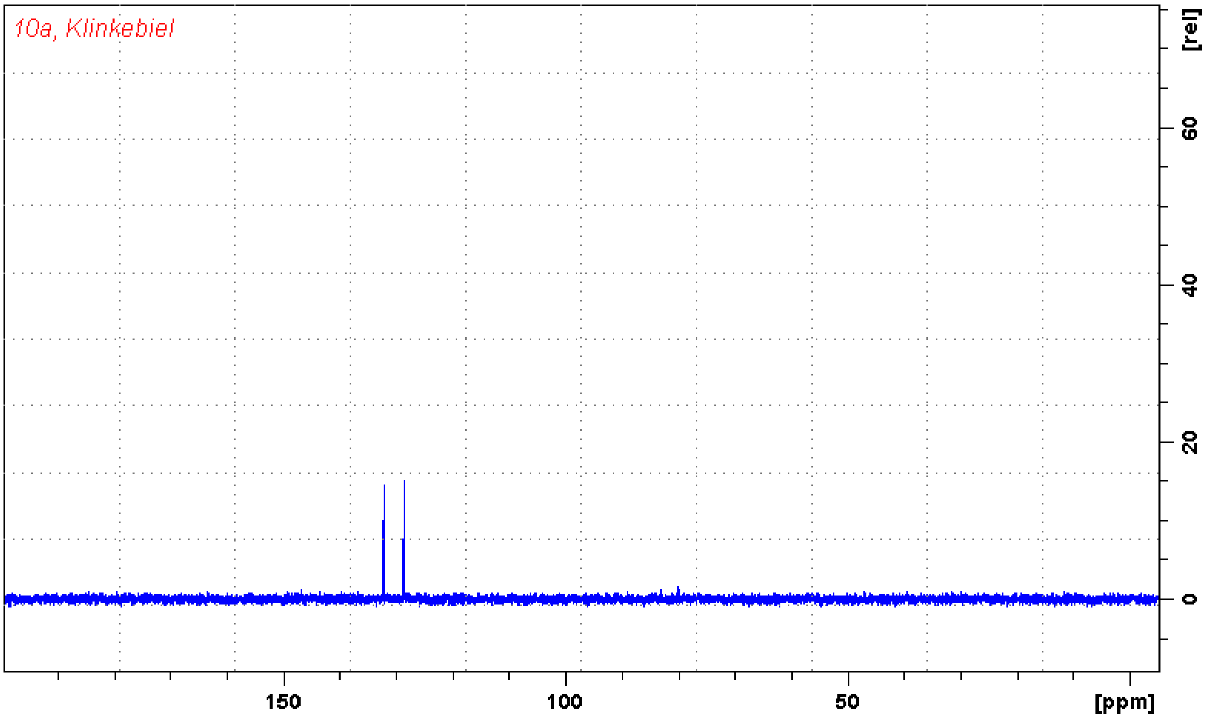

Figure S29. ^{13}C NMR spectrum of **10a**.

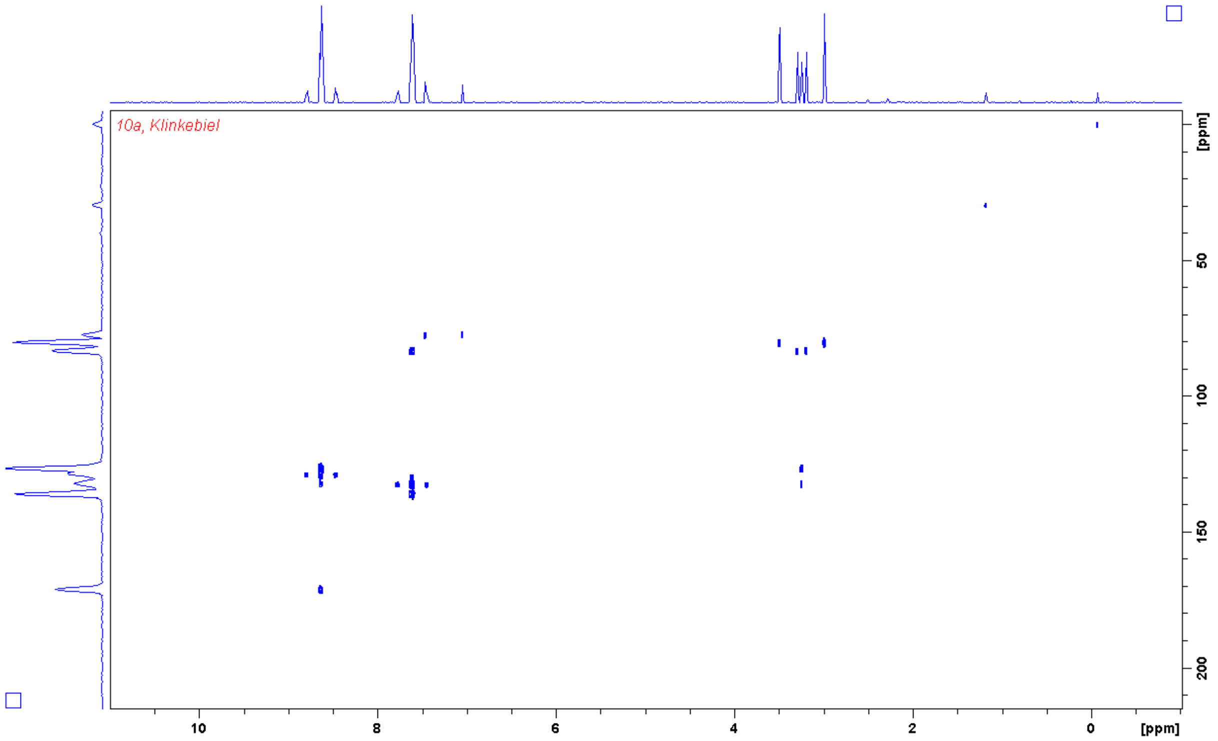

Figure S30. HMBC NMR spectrum of **10a**.

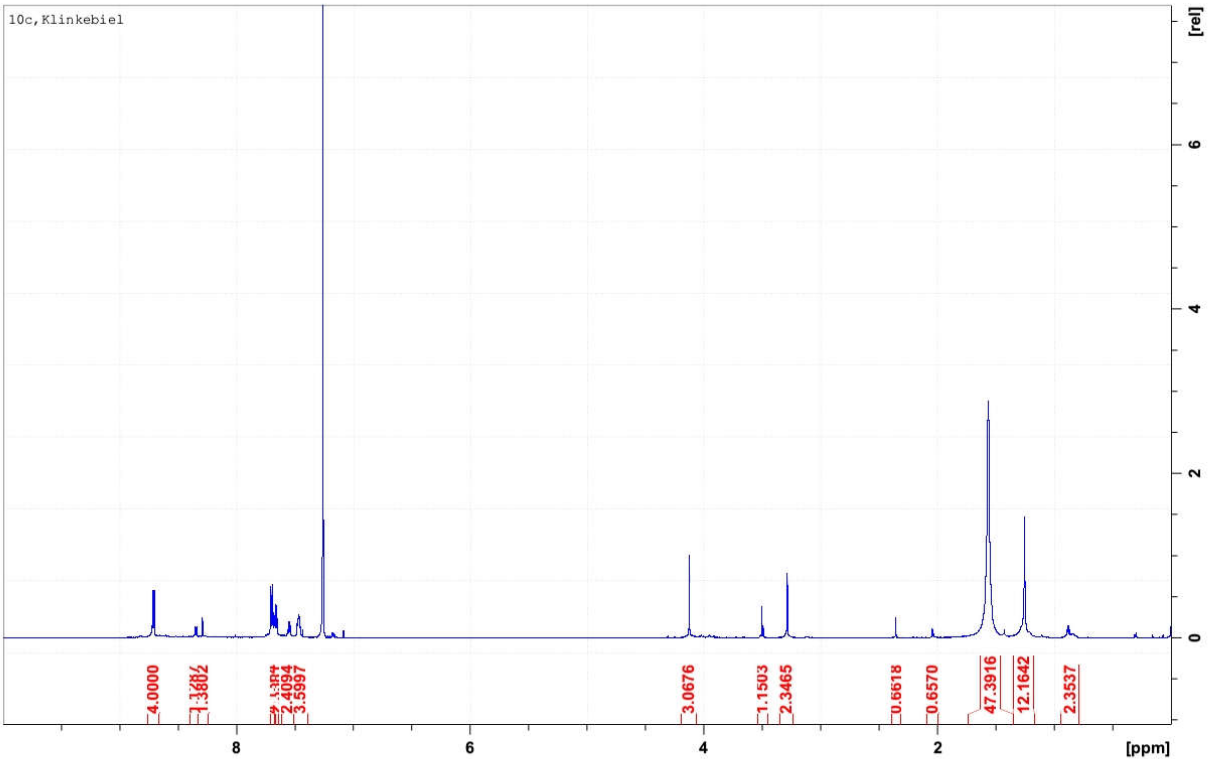

Figure S31. ^{1}H NMR spectrum of **10c**.

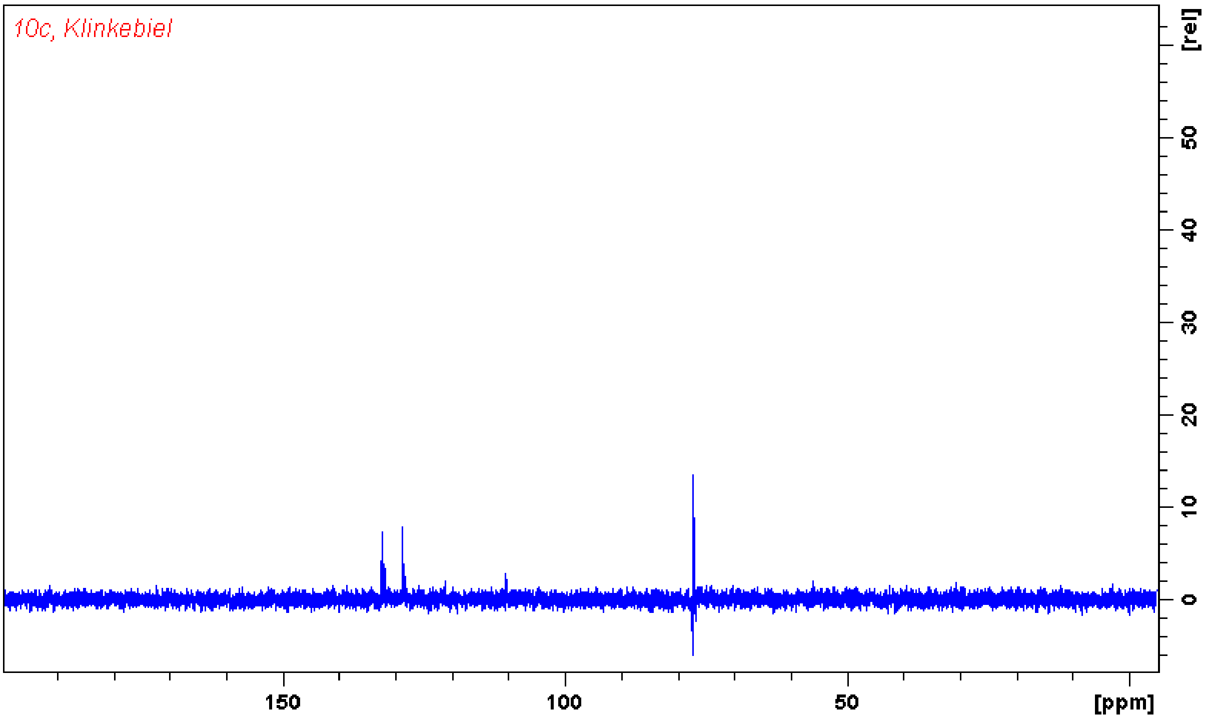

Figure S32. ^{13}C NMR spectrum of **10c**.

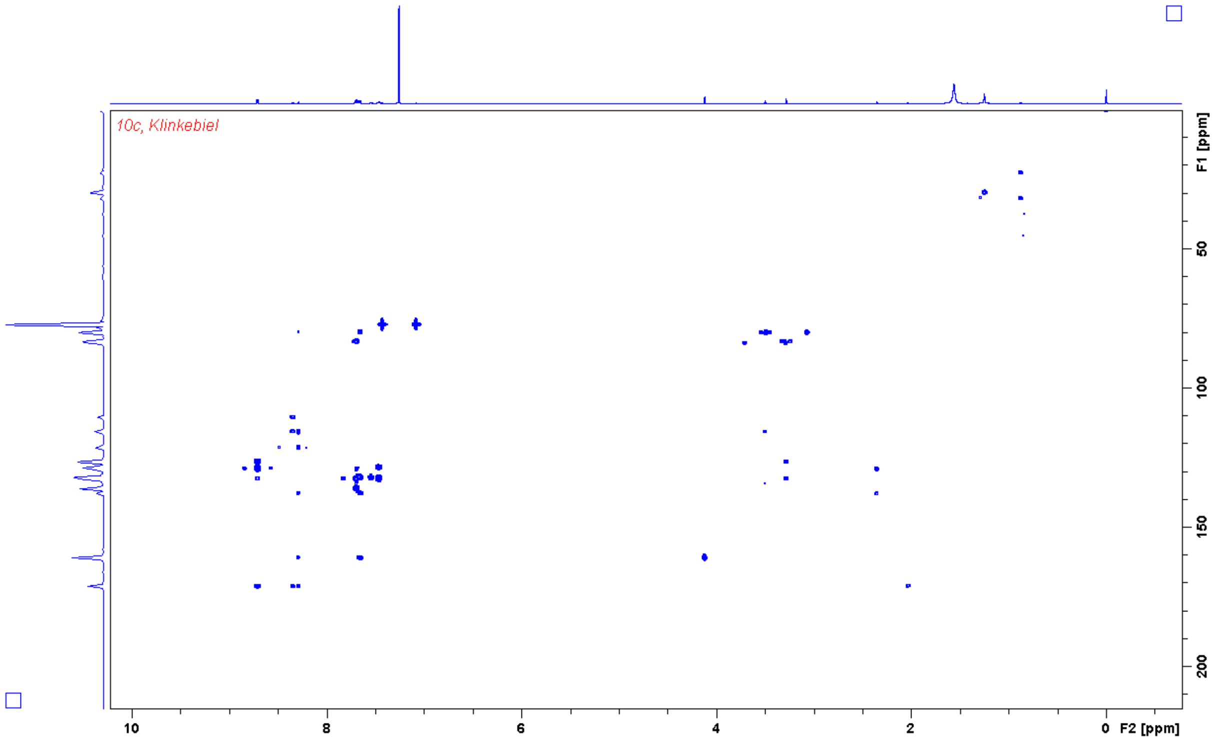

Figure S33. HMBC NMR spectrum of **10c**.

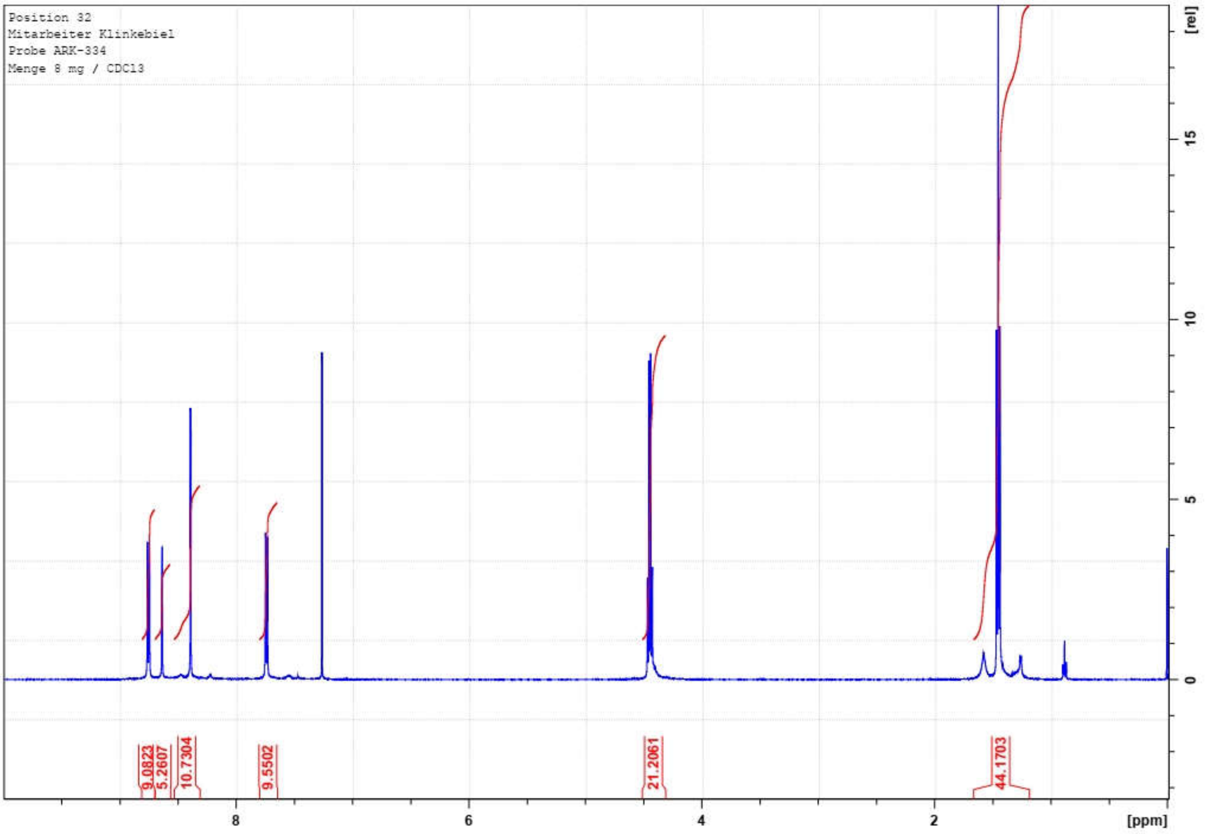

Figure S34. ^{1}H NMR spectrum of **11a**.

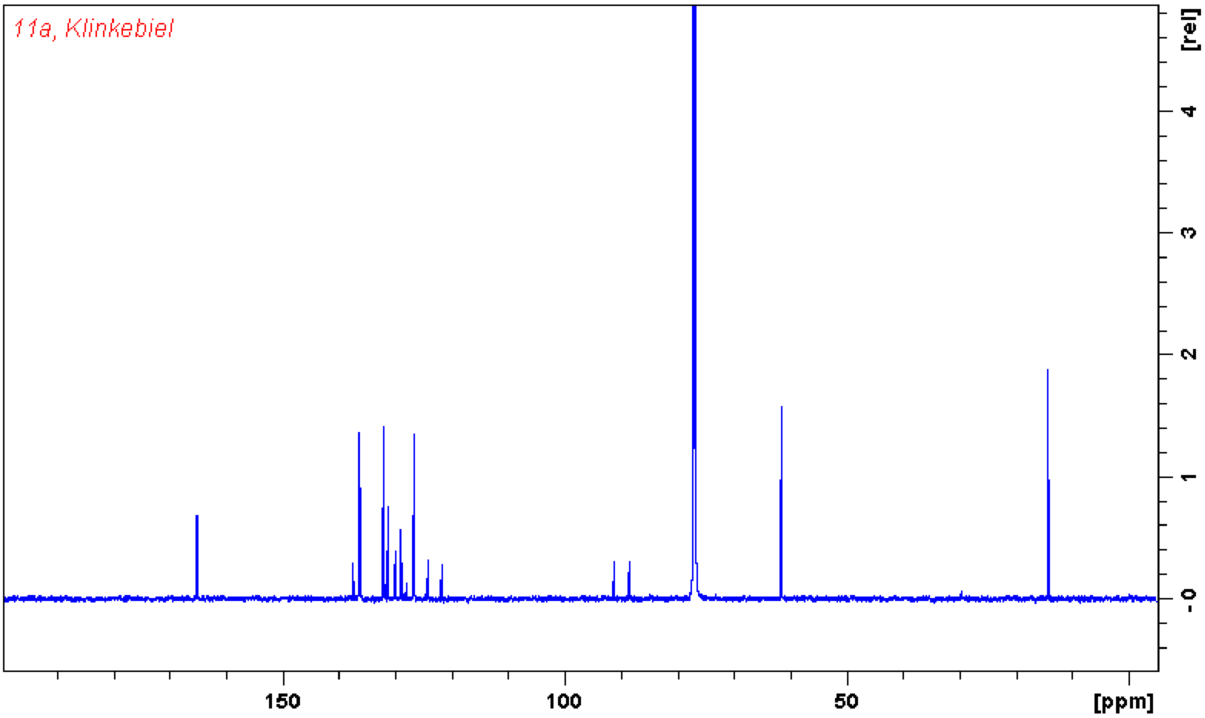

Figure S35. ^{13}C NMR spectrum of **11a**.

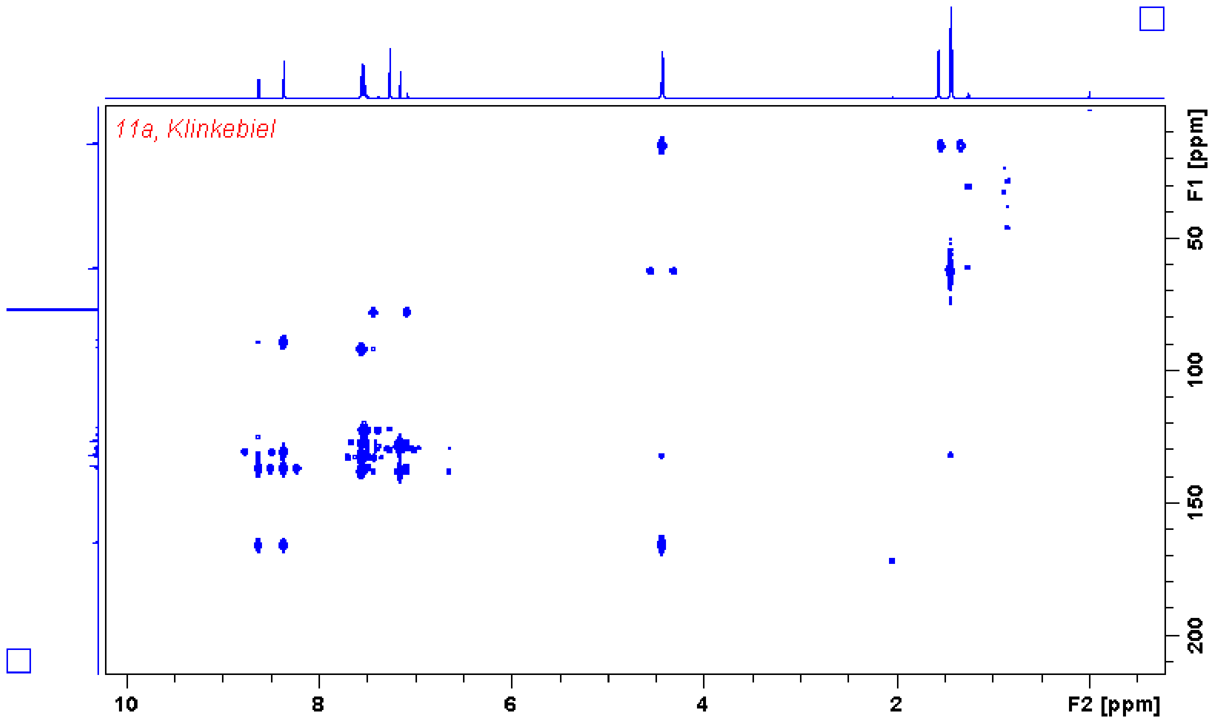

Figure S36. HMBC NMR spectrum of **11a**.

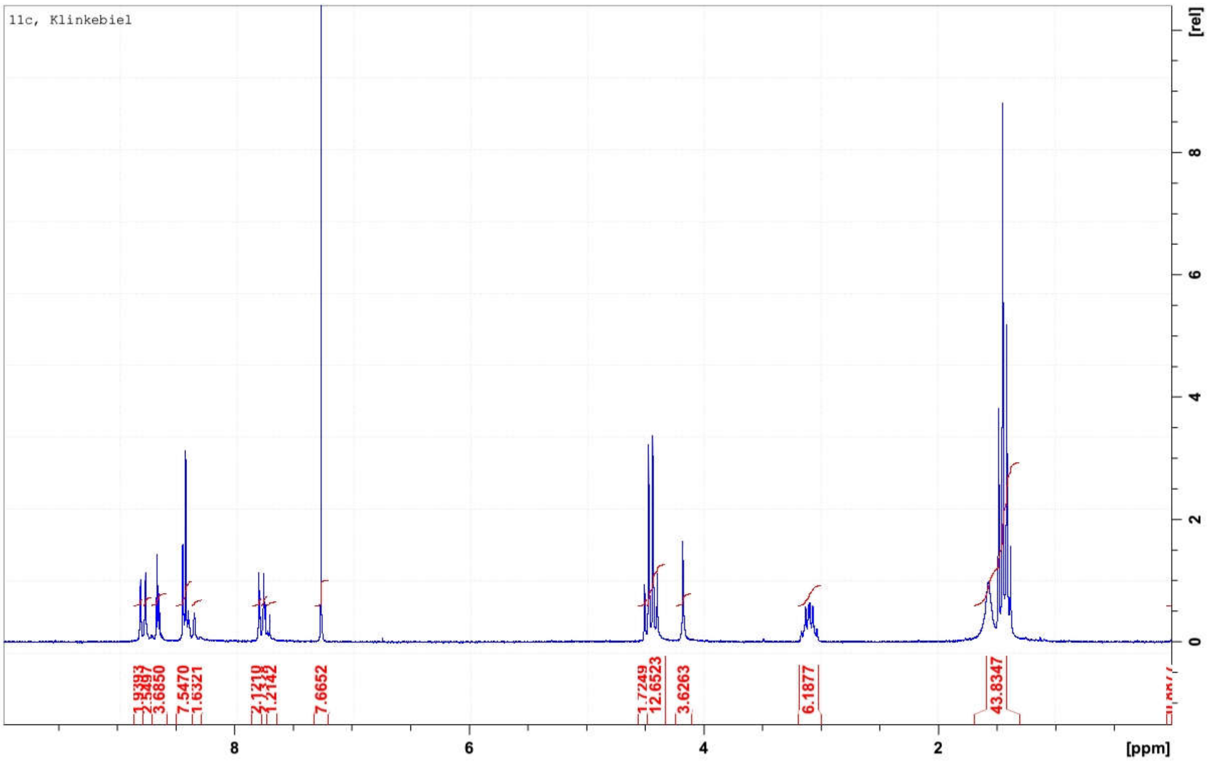

Figure S37. ^{1}H NMR spectrum of **11c**.

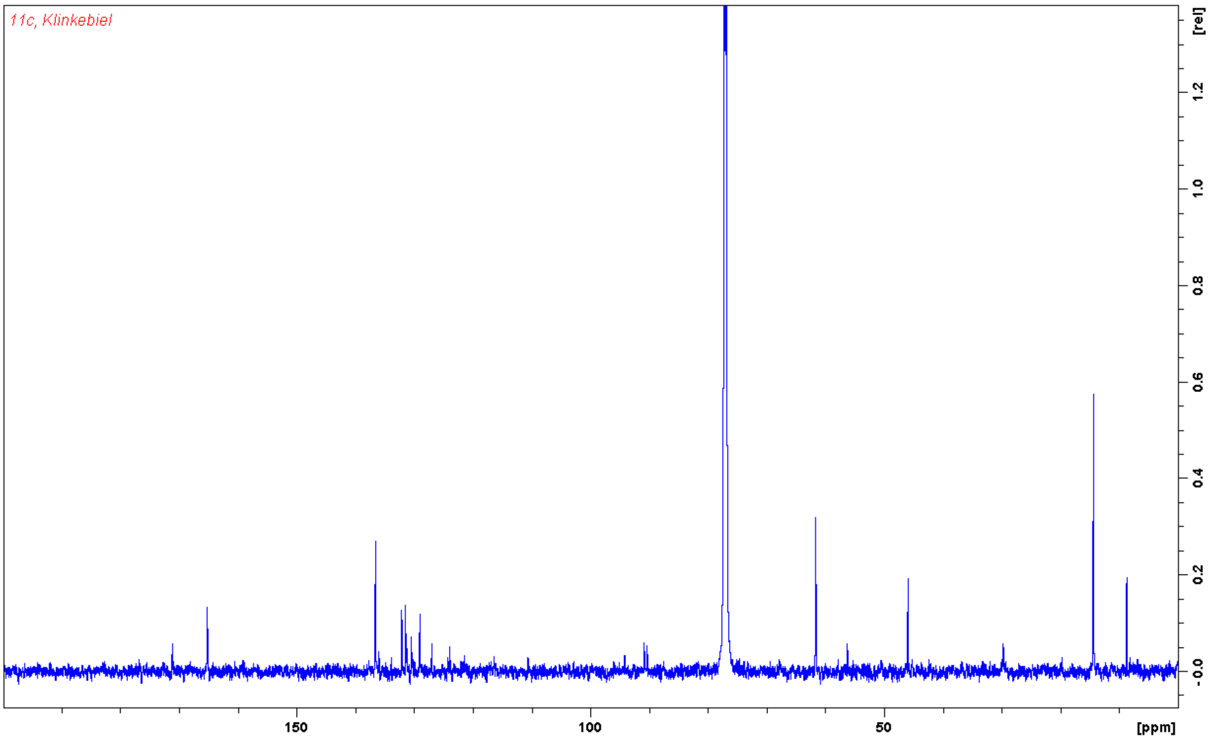

Figure S38. ^{13}C NMR spectrum of **11c**.

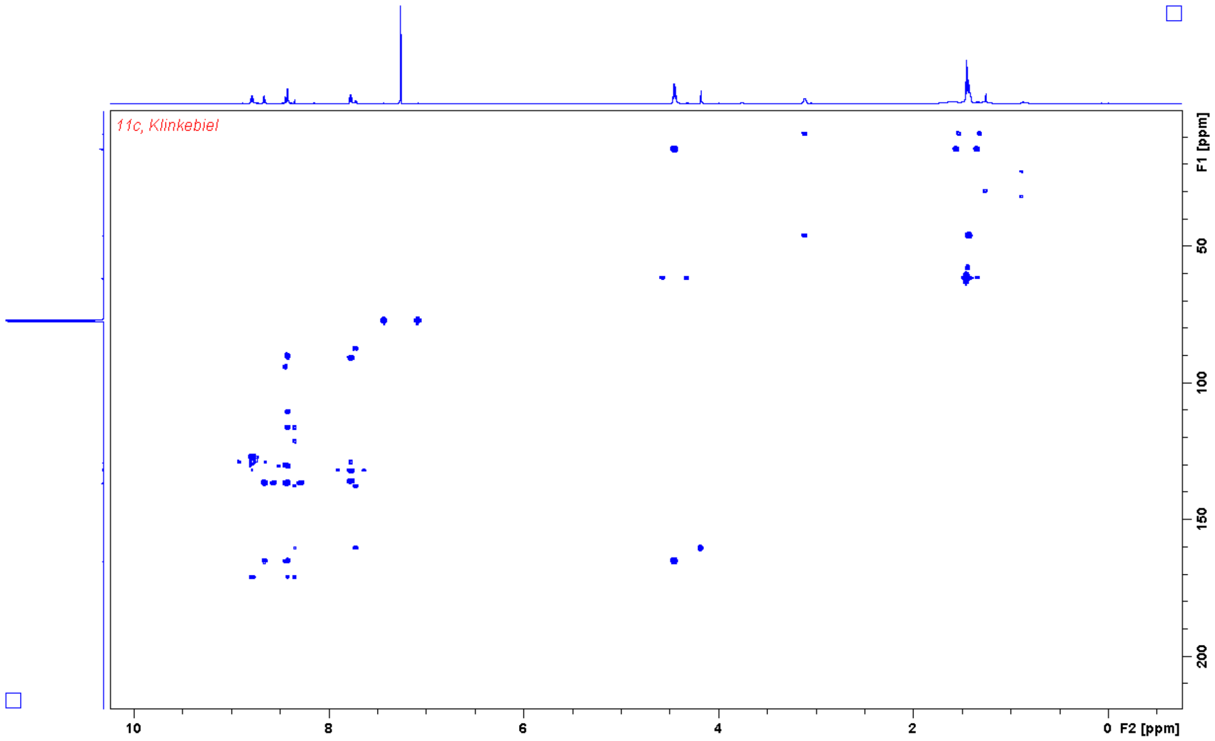

Figure S39. HMBC NMR spectrum of **11c**.
